# Comparison of fractal and grid electrodes for studying the effects of spatial confinement on dissociated retinal neuronal and glial behavior

**DOI:** 10.1038/s41598-022-21742-y

**Published:** 2022-10-20

**Authors:** Saba Moslehi, Conor Rowland, Julian H. Smith, Willem Griffiths, William J. Watterson, Cristopher M. Niell, Benjamín J. Alemán, Maria-Thereza Perez, Richard P. Taylor

**Affiliations:** 1 Physics Department, 1371 University of Oregon, Eugene, OR 97403 USA; 2 Materials Science Institute, 1252 University of Oregon, Eugene, OR 97403 USA; 3 Department of Biology, 1210 University of Oregon, Eugene, OR 97403 USA; 4 Institute of Neuroscience, 1254 University of Oregon, Eugene, OR 97403 USA; 5 Oregon Center for Optical, Molecular and Quantum Science, 1274 University of Oregon, Eugene, OR 97403 USA; 6 Phil and Penny Knight Campus for Accelerating Scientific Impact, 1505 University of Oregon, Franklin Blvd., Eugene, OR 97403 USA; 7 Division of Ophthalmology, Department of Clinical Sciences Lund, Lund University, 221 84 Lund, Sweden; 8 NanoLund, Lund University, 221 00 Lund, Sweden

**Keywords:** Tissue engineering, Gliogenesis

## Abstract

Understanding the impact of the geometry and material composition of electrodes on the survival and behavior of retinal cells is of importance for both fundamental cell studies and neuromodulation applications. We investigate how dissociated retinal cells from C57BL/6J mice interact with electrodes made of vertically-aligned carbon nanotubes grown on silicon dioxide substrates. We compare electrodes with different degrees of spatial confinement, specifically fractal and grid electrodes featuring connected and disconnected gaps between the electrodes, respectively. For both electrodes, we find that neuron processes predominantly accumulate on the electrode rather than the gap surfaces and that this behavior is strongest for the grid electrodes. However, the ‘closed’ character of the grid electrode gaps inhibits glia from covering the gap surfaces. This lack of glial coverage for the grids is expected to have long-term detrimental effects on neuronal survival and electrical activity. In contrast, the interconnected gaps within the fractal electrodes promote glial coverage. We describe the differing cell responses to the two electrodes and hypothesize that there is an optimal geometry that maximizes the positive response of both neurons and glia when interacting with electrodes.

## Introduction

There is growing interest in gaining a fundamental understanding of artificial devices designed to interact with cells of the human nervous system. When implanted in the body, these devices have potentially important applications for the diagnosis and treatment of many neurodegenerative diseases, with retinitis pigmentosa and age-related macular degeneration serving as common examples for the visual system^1–7^. For devices featuring electrodes that stimulate neurons, the electrode design must also accommodate interactions with glia. Although neurons and glia were discovered around the same time, research of the latter has been slower to gain momentum^8^ even though they are prevalent in the central nervous system^9^ and play central roles in controlling neuronal network structure and functionality^10^. In addition to enhancing the performance of medical devices, exploring the differences between the responses of these two cell types to electrodes can be used to investigate fundamental retinal cell behavior and the degree to which their behavior can be controlled.

Strategies to control the presence of glia should balance their positive and negative impacts. Inflammatory as well as other responses from glia can be triggered by the insertion of implants and their micro-motions against the nerve tissue^11–15^ along with mismatches of their mechanical properties (such as rigidity) with the tissue^16,17^. These effects can build glial ‘scars’ that separate the electrode from the targeted neurons and degrade its stimulating power. On the other hand, techniques aimed at completely eliminating glia will have negative long-term impacts on neuronal survival, health, and electrical activity^18^. This is because glia serve as the life support system of neurons, provide natural physical cues for their migration^19–21^, and help to regulate their function^22^, maintain their health^23^, and enhance their synaptic efficacy^24^.

Strategies to control glial responses can be implemented simultaneously to create the least invasive implant. These include decreasing implant size^25^, reducing mechanical mismatches^17^, enhancing surface porosity^26,27^, and covering the implant with a biomimetic or bioactive coating to potentially conceal it from the foreign-body response^13^. Modifications to the physical structure of the surface, for example by introducing nano-roughness or micro-contact printing using lithography, have also been used to control cell attachment and guidance^28–30^ for many purposes, including reducing gliotic responses^31^.

A diverse range of nano/micro-patterned structures have been investigated for their interactions with glial and neuronal cells^27,29,31–36^. Neuronal growth is enhanced by soft, textured surfaces^37–40^ through their close resemblance to the extracellular matrix (ECM) structure^41,42^. In contrast, glial coverage due to cell growth and division is dampened on textured as well as softer substrates as a result of weakened surface interactions^17,32,43–47^. Consistent with these results, an experiment employing a co-culture of cells demonstrated that neurons accumulate on rows of nanowires while glia accumulate in the flat regions between them^31^. Despite all these efforts, the mechanisms controlling the interactions of different cell types with various surfaces are yet to be fully understood.

Our study focuses on the importance of electrode geometry in combination with the electrode material properties for controlling the glial response and associated neuronal behavior. We employ pristine carbon nanotubes as the conducting material of the electrode based on our previous tests with retinal neurons and glia^48^. Furthermore, previous studies have highlighted their ability to decrease glial scar tissue formation^49^ and have demonstrated their capacity to stimulate neurons effectively^50–52^ and boost signal transmission^53–56^.

By growing vertically-aligned carbon nanotubes (VACNTs) on a silicon dioxide (SiO_2_) substrate, we generate laterally-patterned regions of textured electrodes (VACNT) and smooth gaps (SiO_2_). Based on the above studies of cell responses to differing textures, the VACNT regions are designed to predominantly accumulate neurons while the smooth SiO_2_ regions accumulate glia. Through this design, the VACNT regions serve as the scaffold for the neurons normally provided by the glia in the retina. By accumulating in the gaps of this scaffold, the glia will be sufficiently close to provide trophic and metabolic support to the neurons on the electrodes without physically blocking the neuron-electrode interactions necessary for maximum stimulation. Approximately 25 µm high, the VACNT electrodes act as barriers to the glia and therefore present the potential to guide glial coverage across the SiO_2_ surface. Our study investigates the impact of confinement on cell behavior by considering a ‘closed’ system, in which the VACNT electrodes form an array of gaps which are disconnected from each other by the restricting walls, and an ‘open’ system in which the electrodes are surrounded by connected gaps.

Rather than conducting a comprehensive study of various ‘closed’ and ‘open’ designs, here we focus on two examples that are suitable for electrode applications and demonstrate some fundamental cell behaviors that are useful for informing future electrode designs. With this goal, grid and fractal electrodes serve as appealing examples of these two designs. Both have previously been considered for electrodes that interface with neurons and/or glia^57–65^. For each geometry, the VACNTs form continuous networks that facilitate electrode biasing (Supplementary Information). In addition, the large electrical capacitances generated by their geometries are predicted to enhance neuron stimulation^57^ and both allow transmission of light through their gaps. However, these properties are only beneficial if accompanied by favorable cell responses. Our study of grids builds on a pioneering investigation from 1978 showing that glia generated from a human brain biopsy could be confined within the chambers of a metallic grid pattern^65^. This ‘closed’ system featured an array of disconnected chambers ~ 100 µm in width. Here we fabricate similar-sized chambers of 60 µm width, which are sufficiently large to accommodate several glial cell bodies and processes. However, whereas the original study featured a culture of only glia, we employ a co-culture to allow the investigation of the interactions between the glia and neurons. The VACNT electrode width is chosen to be *W*_*CNT*_ = 20 µm. This width allows the somas of several neurons to attach to the VACNT surfaces and for their processes to grow out from the somas across the electrode surface.

**Figure 1:**
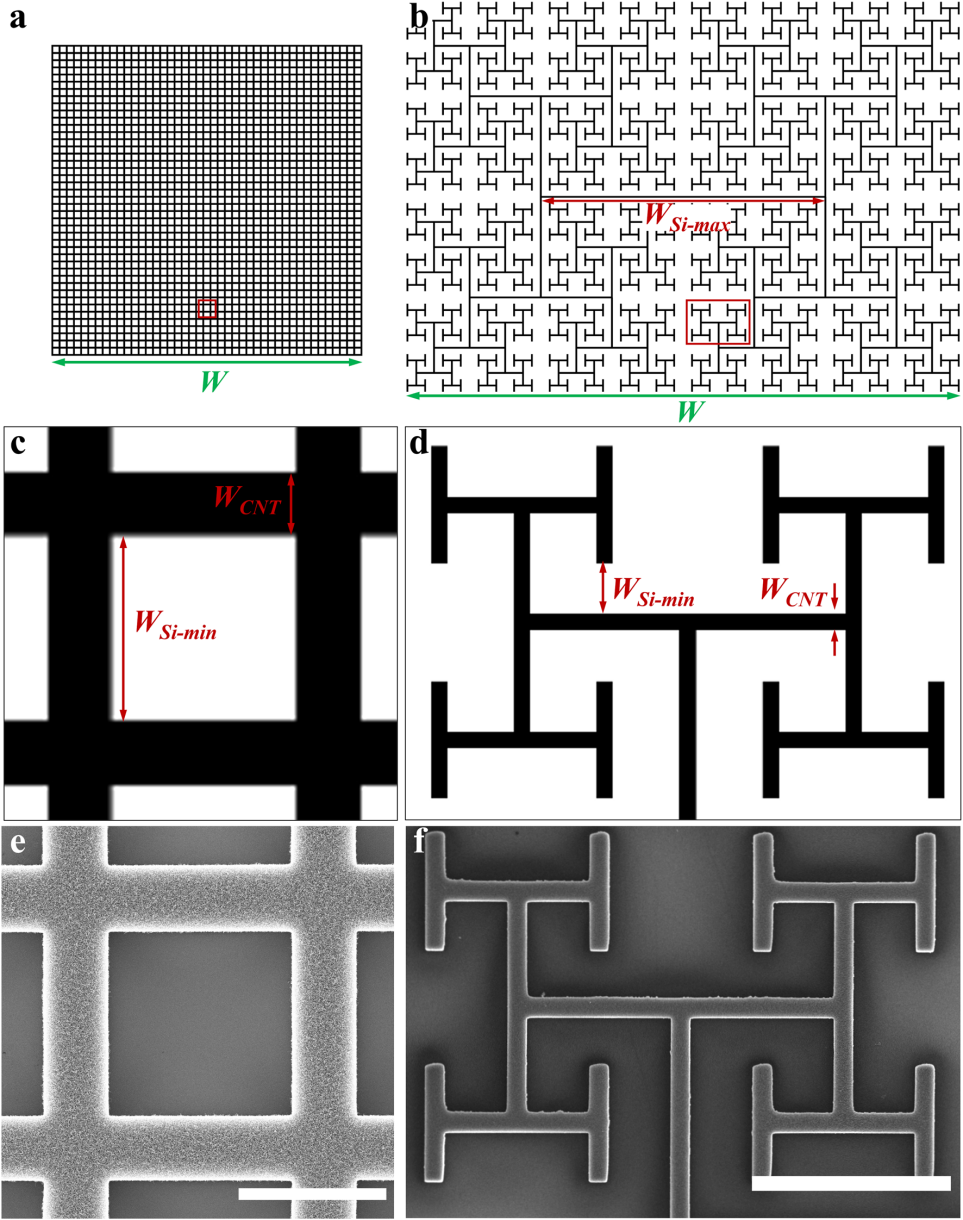
The lithography mask designs and scanning electron microscopy (SEM) images of the grid and fractal electrodes. (**a**,**b**) Binary masks of the grid and fractal electrodes respectively. (**c**,**d**) the zoom-in of the red boxes in (**a**) and (**b**) showing some of the geometric parameters of the two electrodes (see “Materials and methods” section and Supplementary Information for definitions). (**e**,**f**) SEM images of VACNTs in a region equivalent to the red boxes in (**a**) and (**b**) for the grid and fractal electrodes, respectively. The mask sizes in (**a**) and (**b**) are to scale (see Table 1 for relative sizes). Scale bars in (**e**) and (**f**) are 50 and 200 µm, respectively.

In terms of our choice of the ‘open’ system, fractal branches are prevalent in nature^66–68^. In addition to plants, trees^69^, and rivers^70^, bronchial^71^, cardiovascular^69^, and neural networks^72^ all feature fractal branches. Their branches spread out in space, producing two embedded fractal patterns—the branches and the gaps forming between them—and their structural relationship can lead to enhanced functionality. In particular, the multi-scaled gaps form an interconnected system that interfaces efficiently with the penetrating branches. Whereas, for example, this gap-branch interplay facilitates light exposure for trees^73^ and oxygen transfer for bronchial trees^74^, a recent study conducted by the authors investigated glial-neuron interactions with fractal electrodes fabricated from VACNTs^59^. Our electrodes feature an ‘H-Tree’ geometry that repeats at multiple scales (Fig. 1). To facilitate a comparison with the grids, the VACNT electrode width is chosen to be 20 µm and the smallest gap width (see Supplementary Information) of 60 µm matches the chamber width of the grid electrodes. This size allows glia to connect to the increasingly larger gaps within the fractal electrode which can be viewed as a set of interconnected chambers.

We consider in vitro experiments using a co-culture of dissociated mouse retinal neurons and glia because it provides a controlled environment in which fluorescence microscopy can be used to examine cell behavior and cell-electrode interactions as they evolve over 17 days in vitro (DIV). Using qualitive and quantitative observations, we find that the grid electrodes trap the glia inside the disconnected chambers, preventing them from covering the smooth SiO_2_ regions. In contrast, the fractal electrodes provide the glia with the opportunity to divide and grow and so gradually cover increasingly larger connected SiO_2_ regions. While both electrodes support adhesion, growth, and survival of the neurons, we find that the grid electrodes promote the greatest neuron process density on both the VACNT and SiO_2_ surfaces when compared to the fractal electrodes. Their ‘closed’ geometry increases the proximity of the neurons to the interface edges between the two surfaces. These edges serve as anchor points for neuron somas and encourage their processes to grow and connect to other neurons. We will discuss the consequences of this interplay of the two cell types for cell health and stimulation.

## Materials and methods

### Electrode design parameters

We fabricated H-Tree geometries based on the number of repeating levels being *m* = 5 and a fractal dimension of *D* = 2 which sets the rate of shrinkage between these levels (see Supplementary Information for a more detailed explanation of *m*,* D*, and the related geometric parameters discussed in this section)^59^. When coupled with *W*_*CNT*_ = 20 µm and *W*_*Si-min*_ = 60 µm, these parameters generate a maximum electrode separation distance of *W*_*Si-max*_ = 3.22 × 10^3^ µm and an overall electrode width of *W* = 6.26 × 10^3^ µm (Table 1, Supplementary Fig. S1). The *W*_*CNT*_ for the grids was set equal to the fractals and their overall electrode width was set to *W* = 3.51 × 10^3^ µm (corresponding to 43 chambers per row) to provide approximately the same total edge length, *E*, and approximately the same covering area, *A*_*CNT*_, as the fractal electrode. *E* was standardized because it represents the interaction boundary between the neuron-rich electrodes and glia-rich gaps. *A*_*CNT*_ was also standardized to ensure similar amounts of VACNT material as well as similar cross-sectional areas for both electrodes during initial interactions with the cells. We note that, due to the two electrodes belonging to fundamentally different geometries (multi-scaled fractals versus uni-scaled ‘Euclidean’ grids), it is not possible to control for all of their parameters—consequently, the two electrodes differ in their bounding areas, *A*_*bounding*_, and SiO_2_ surface areas, *A*_*Si*_. We also note that all fractal geometric parameters were calculated as a function of *D*, *m*, *W*, and *W*_*CNT*_, and therefore are interdependent. Table 1 summarizes the various parameters of the two electrode designs.

**Table 1:** Geometric parameters for the grid and fractal electrodes.

Group	*W*_*CNT*_ (µm)	*W*_*Si-min*_ (µm)	*W*_*Si-max*_ (µm)	*W* (µm)	*E* (µm)	*A*_*CNT*_ (µm^2^)	*A*_*Si*_ (µm^2^)	*A*_*Si*_*/A*_*CNT*_	*A*_*bounding*_ (µm^2^)
Grid	20	60	60	3.51 × 10^3^	4.66 × 10^5^	5.36 × 10^6^	6.91 × 10^6^	1.28	1.23 × 10^7^
Fractal	20	60	3.22 × 10^3^	6.26 × 10^3^	4.62 × 10^5^	4.62 × 10^6^	2.31 × 10^7^	5.88	2.77 × 10^7^

### Electrode synthesis and characterization

Microfabrication and lithography techniques were used to synthesize the VACNT fractal and grid electrodes following procedures that have been described in detail elsewhere^48^. Briefly, 2-inch silicon wafers with a 300 nm thermal oxide (SiO_2_) top layer were cleaned and patterned using photolithography techniques. The whole wafer contained 10 individual grid and 8 individual fractal electrodes. After photoresist development, a 2–5 nm aluminum (Al) adhesive layer was deposited thermally followed by an electron-beam deposition of a 3–5 nm iron (Fe) catalyst layer. After 30 s of acetone soaking accompanied by sonication to lift off the photoresist layer, the wafer was then cut into individual samples with each sample featuring one electrode. Not all of the fabricated samples were used in the dissociated cell culture experiments. Some samples were eliminated due to visible defects or were saved for further SEM characterization. Chemical vapor deposition (CVD) techniques were used to synthesize VACNTs on the catalyst patterns in a 2-inch quartz tube. A 2:1 mixture of ethylene (C_2_H_4_):hydrogen (H_2_) (200 and 100 SCCM respectively) accompanied by a 600 SCCM flow of Argon (Ar) was maintained during the 3-min growth time at 650 °C. This technique resulted in patterned electrodes consisting of entangled ‘forests’ of VACNTs (Fig. 1, Supplementary Fig. S2). The electrodes were then stored in integrated circuit trays in a desiccator cabinet. The top surface and sidewalls of the VACNTs, their heights and general conditions were inspected using a ZEISS-Ultra-55 scanning electron microscope. No visual differences were observed between samples from the two electrode designs and fabrication runs. VACNT heights were in the range of 16–36 µm. During culture, the samples were placed in 4-well culture plates (Sarstedt, Newton, NC) with one sample per well.

### Dissociated retinal cell cultures

Wildtype C57BL/6J mice were kept at animal welfare services at University of Oregon (UO) with full time access to fresh water and food supplies. Handling and culture procedures involving the mice were performed according to protocols approved by the UO’s Institutional Animal Care and Use Committee (IACUC) under protocol 16-04, in compliance with the ARRIVE guidelines and National Institutes of Health guidelines for the care and use of experimental animals. Dissociated retinal cell cultures were employed using protocols described elsewhere^31,48,75^. Briefly, postnatal day 4 (PN4) mice were euthanized by decapitation and their retinas quickly dissected and kept in Dulbecco’s Modified Eagle Medium (DMEM—ThermoFisher Scientific, Waltham, MA) containing high-glucose, sodium pyruvate, l-glutamine, and phenol red. For each culture experiment, four retinas were transferred into an enzyme solution containing DMEM, papain (Worthington Biochemical Corporation, Lakewood, NJ) and l-cysteine (Sigma-Aldrich, St Louis, MO). The digested retinas were carefully rinsed with DMEM and transferred to new DMEM containing B27 (Sigma-Aldrich, St Louis, MO) and l-glutamine–penicillin–streptomycin (Sigma-Aldrich, St Louis, MO). The dissociated retina solution was centrifuged and the cell pellet was re-suspended in the DMEM/B27/antibiotic solution. The cell suspension (500 µL) was subsequently seeded onto each well containing either a grid or a fractal electrode. The cells were cultured for 17 DIV at 37 °C and 5% CO_2_. The culture medium was first changed at 3 DIV and then every other day until the end of the culture time. No protocols such as precoating the surfaces with poly-d-lysine (PDL) or poly-l-lysine (PLL) were used to increase the neuronal and glial adhesion to the different surface types. The live cell density as measured by a hemocytometer was (3.7 ± 0.4) × 10^6^ cells/mL.

### Immunocytochemistry

The immunocytochemistry protocol is described in detail elsewhere^31,48,75^. Briefly, the cells were fixed with 4% paraformaldehyde (PFA), rinsed with a phosphate buffered solution (PBS) and pre-incubated in PBS-complete, containing PBS, Triton-X (Sigma-Aldrich, St Louis, MO), bovine serum albumin (BSA) (Sigma-Aldrich, St Louis, MO), goat normal serum and donkey normal serum (Jackson ImmunoResearch, West Grove, PA). The cells were subsequently incubated with PBS-complete containing the primary antibodies, mouse anti-β-tubulin III (neuronal marker for several neuron types in the mouse retina^76,77^—Sigma-Aldrich, St Louis, MO) and rabbit anti-glial fibrillary acidic protein (GFAP; glia marker—Agilent, Santa Clara, CA) over night at 4 °C. The cells were then rinsed and incubated with PBS-complete containing the secondary antibodies Cy3 goat anti-mouse IgG and AlexaFluor 488 donkey anti-rabbit IgG (Jackson ImmunoResearch, West Grove, PA). The secondary antibody solution was then removed and the cells rinsed with PBS. The samples were transferred to microscope slides and mounted with Vectashield containing DAPI (fluorescent cell nuclear marker that binds to DNA—Vector Laboratories, Burlingame, CA).

### Fluorescence microscopy

A Leica DMi8 inverted fluorescence microscope was used to take 20× images in the Cy3 (excited at 550 nm, emission peak at 570 nm), AlexaFluor 488 (excited at 493 nm, emission peak at 519 nm) and DAPI (excited at 358 nm, emission peak at 461 nm) channels for all electrodes. The top VACNT and bottom SiO_2_ surfaces were imaged separately with the focus being adjusted to these surfaces. The 2048 × 2048 pixel^2^ (662.65 × 662.65 µm^2^) field of views (FOVs) in each channel were then stitched together using an automated stitching algorithm with 10% overlap at the edges of neighboring FOVs to create full electrode images.

### Quantitative measurement of the neuron process length and glial coverage

We chose neuron process growth as the morphological phenotype measurement of neuronal cell health and function^78,79^. This was in part based on the long-term goal of employing electrodes for neuron stimulation and the high density of stimulation sites on the processes. The quantitative analysis involved a calculation of the density of processes (i.e. total length of the neurons’ dendrites and axons within a given surface area). Based on their role of promoting neuron homeostasis and survival, the glial analysis focused on quantifying the surface area expressing the cytoskeletal marker GFAP^32^. This analysis involved a calculation of their surface coverage density (referred to hereafter as ‘coverage’, i.e. the surface area covered by glia normalized to the total surface area available).

To perform this quantitative analysis^59^, binary masks were created using a MATLAB algorithm for the fractal and grid electrodes based on the *W*_*CNT*_ measured using the DAPI channel of the fluorescence images. These masks were then applied to all acceptable FOVs for each electrode so that the SiO_2_ and VACNT surfaces could be analyzed separately. Unacceptable FOVs (e.g. those with any abnormalities such as VACNT deformations) were rare: typically 2 out of 50 FOVs. An automated image analysis MATLAB algorithm was integrated with the binary mask algorithm to detect and measure the process length per FOV on the SiO_2_ and VACNT surfaces separately. In cases when processes physically overlapped (for example, when they ‘bundled’ together and followed a common route on the surface or when multiple processes followed the same electrode edge), the algorithm detected these as one process. This resulted in an undercount of processes, especially on the VACNT surfaces, but did not affect the general results of the experiments. For each electrode, the normalized process length on the SiO_2_ (*N*_*Si*_) and the VACNT (*N*_*CNT*_) surfaces was then defined as the total process length on each surface across all FOVs (*N*_*LSi*_ or *N*_*LCNT*_) divided by the total area of that surface in the electrode (*A*_*Si*_ or *A*_*CNT*_):

1 $${N}_{Si}= \frac{{N}_{LSi}}{{A}_{Si}}$$

2 $${N}_{CNT}= \frac{{N}_{LCNT}}{{A}_{CNT}}$$

For the glia, a semi-automated thresholding MATLAB algorithm integrated with the binary mask algorithm was used to detect and measure the glial area per FOV on the SiO_2_ and VACNT surfaces separately. For each electrode, the normalized glial area on the SiO_2_ and the VACNT surfaces were then defined as the total glial area on each surface across all FOVs (*G*_*ASi*_ or *G*_*ACNT*_) divided by the total area of that surface:

3 $${G}_{Si}= \frac{{G}_{ASi}}{{A}_{Si}}$$

4 $${G}_{CNT}= \frac{{G}_{ACNT}}{{A}_{CNT}}$$

To minimize the error in detecting neuron process length and glial area around the edges of the electrodes on both surfaces, FOVs were inspected and mask measurements were adjusted manually if necessary to allow for the correct detection of in-focus features (see Supplementary Fig. S3 for examples of glial coverage and neuron process detection algorithms on the VACNT surface of fractal electrodes).

### Statistical analysis

The Shapiro–Wilk test was performed to determine the normality of the neuronal and glial parameters. Because some of the distributions failed the normality criteria, the nonparametric Kruskal–Wallis comparison for significance (with a significance level of 0.05) was used in MATLAB to compare the medians of neuronal and glial parameters against various null hypotheses (for example *G*_*Si*_ and *G*_*CNT*_ were tested against the null hypothesis that surface material would not impact glial behavior). The outliers were determined as any datapoint that was below the minimum set as Q1-1.5IQR or above the maximum set as Q3 + 3IQR, where Q1, Q3, and IQR represent the 25th% quartile, 75th% quartile, and the interquartile range (Q3–Q1) respectively. A total number of 7 grid electrodes from 3 independent cultures and 11 fractal electrodes from 5 independent cultures were used in the experiments. Some samples were excluded due to complications in fabrication or culturing procedures. Each independent culture included both electrode designs.

## Results

**Figure 2:**
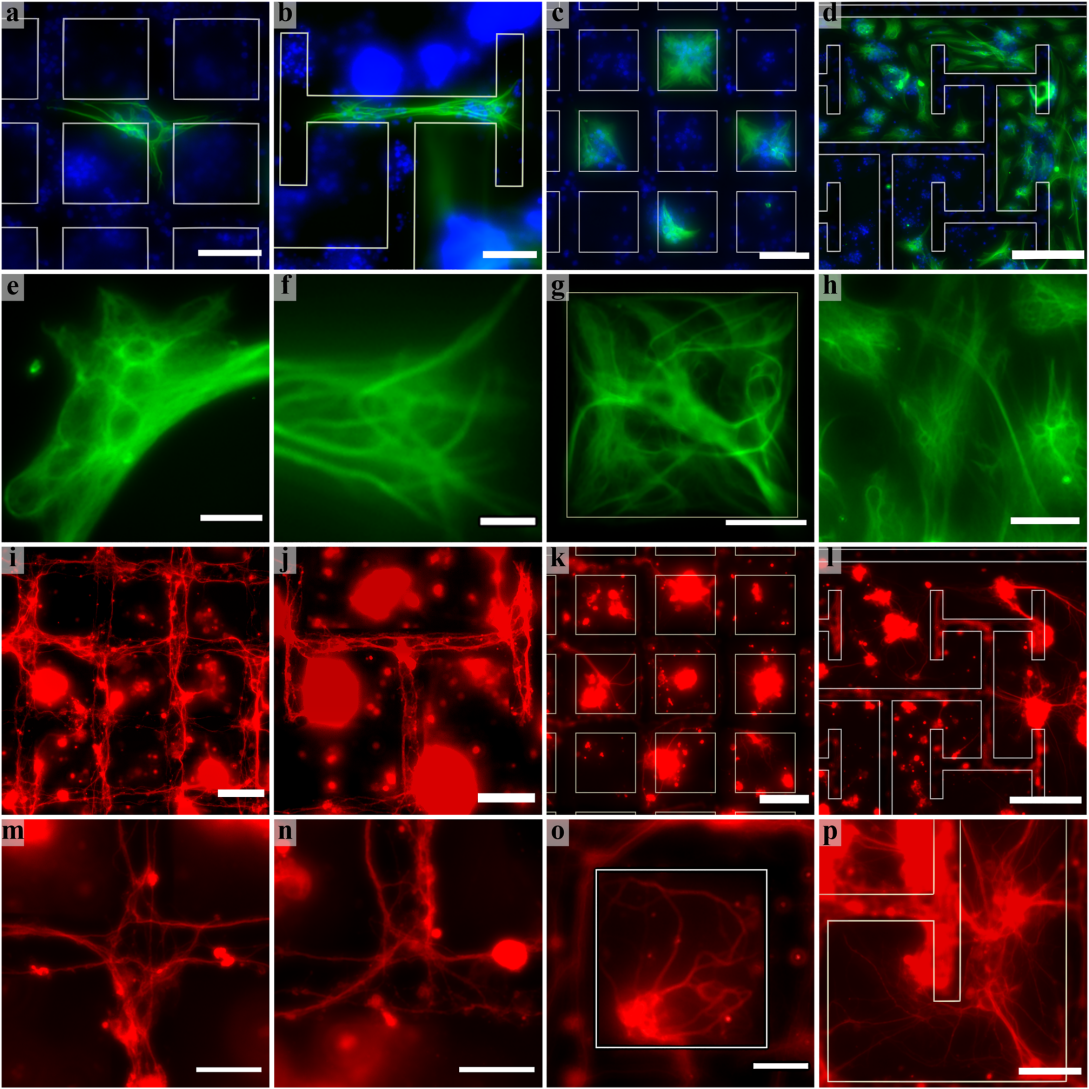
Representative examples of fluorescence images of retinal cells interacting with the grid and fractal electrodes at 17 DIV (green = GFAP labelled glia; red = β-tubulin III labelled neurons; blue = DAPI labeled nuclei). Glia on the VACNT surfaces of the (**a**) grid and (**b**) fractal electrodes. Glia accumulating on the SiO_2_ surfaces of the (**c**) grid and (**d**) fractal electrodes. The structure of glia on the VACNT surfaces of the (**e**) grid and (**f**) fractal electrodes as well as the SiO_2_ surfaces of the (**g**) grid and (**h**) fractal electrodes. Neuron processes following the VACNT electrodes of the (**i**) grid and (**j**) fractal electrodes. (**k**) Neuron clusters inside the grid chambers sending processes towards the VACNT sidewalls. (**l**) Large neuron clusters on the SiO_2_ surface connecting to the neurons on the VACNT surface of a fractal electrode. Neuron processes following the VACNT electrodes of the (**m**) grid and (**n**) fractal electrodes. (**o**) Neuron cluster attached to the VACNT sidewall of a grid chamber sending processes onto both the SiO_2_ and VACNT surfaces. (**p**) Neuron clusters and connecting processes on the SiO_2_ and VACNT surfaces of a fractal electrode. The images in (**c**) and (**k**) show the same FOV, as do (**d**) and (**l**). Electrode edges are highlighted with white lines except for panels (**i**), (**j**), (**m**), and (**n**) which concentrate on the behavior of processes along the edges because the lines would have obscured these processes. Scale bars are: 10 µm in (**e**) and (**f**); 20 µm in (**g**), (**h**), (**m**), (**n**), and (**o**); 40 µm in (**p**); 50 µm in (**a**), (**b**), (**c**), (**i**), (**j**), and (**k**); and 100 µm in (**d**) and (**l**).

First, we focus on some qualitative observations of cell behavior. Figure 2 and Supplementary Fig. S4 show representative fluorescence images of the cell interactions with the electrodes. Glia were observed on both the SiO_2_ and VACNT surfaces of both electrodes at 17 DIV. The glia residing on the SiO_2_ never extended processes over the VACNT surfaces. In terms of morphology, the glia exhibited a spread-out shape with multiple, well-defined long processes on the SiO_2_ surfaces of both electrodes. In contrast, they had a more elongated morphology on the VACNT surfaces of both electrodes. These followed the shape of the VACNT surfaces and, although rare, even made 90° turns at the VACNT turning points.

Neurons adhered to and grew processes on both the SiO_2_ and VACNT surfaces of the fractal and grid electrodes. For both surfaces, neuron somas sometimes clustered together and some of the processes connecting the clusters formed bundles. This behavior was observed more frequently on the SiO_2_ surface than the VACNT surface (Fig. 2i–l). Neurons on the two surfaces were connected via clusters attached to the VACNT sidewalls and processes were frequently observed following the top and bottom edges of the sidewalls (Fig. 2, Supplementary Fig. S4). The large clusters on the SiO_2_ surfaces were much more common for the fractal than the grid electrodes, particularly in regions accompanied by large glial coverage. Large clusters were occasionally evident in some grid chambers and this most often occurred when glia were present. For example, in Fig. 2c and k the cluster in the central chamber (which has no glia) appears to be smaller than those in the surrounding four chambers (which are occupied by glia).

**Figure 3:**
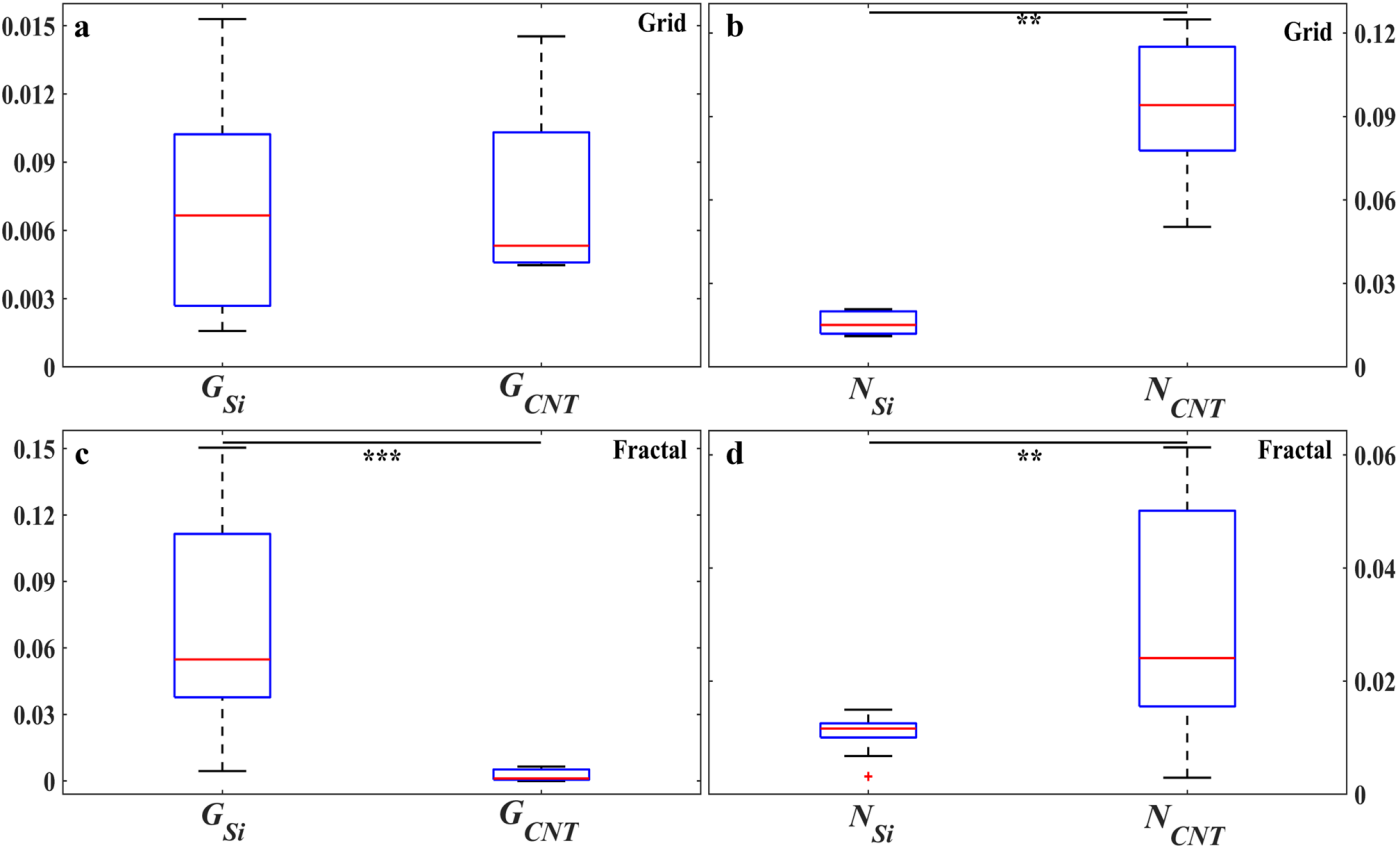
Comparison of glial and neuronal behavior on the SiO_2_ and VACNT surfaces for the grid and fractal electrodes at 17 DIV. Statistical analysis showing boxplots of *G*_*Si*_ (left) compared with *G*_*CNT*_ (right) for the (**a**) grids and (**c**) fractals, as well as *N*_*Si*_ (left) compared with *N*_*CNT*_ (right) for the (**b**) grids and (**d**) fractals. The y axes of (**a**) and (**c**) display the range of *G*_*Si*_ and *G*_*CNT*_ values and the y axes of (**b**) and (**d**) display the range of *N*_*Si*_ and *N*_*CNT*_ values. Stars in panels (**b**–**d**) indicate the degrees of significance: *** and **denote *p* ≤ 0.001 and *p* ≤ 0.01, respectively. The red plus in panel (**d**) is an outlier. Note that *G*_*Si*_ and *G*_*CNT*_ are unitless and *N*_*Si*_ and *N*_*CNT*_ have units of µm^−1^ (see “Materials and methods” section).

We now move to quantitative measures. Firstly, the effect of the SiO_2_–VACNT material system on the glial and neuronal distributions on the two surfaces was studied through a statistical comparison of *G*_*Si*_ versus *G*_*CNT*_ and *N*_*Si*_ versus *N*_*CNT*_ for the grid and fractal electrodes separately. There was no significant difference between *G*_*Si*_ and *G*_*CNT*_ for the grids (Fig. 3a), whereas *G*_*Si*_ was significantly higher for the fractals (*p* = 0.0002, Fig. 3c). *N*_*Si*_ was significantly lower than *N*_*CNT*_ for both the grids and fractals (*p* = 0.0017 and 0.0053 respectively, Fig. 3b,d).

**Figure 4:**
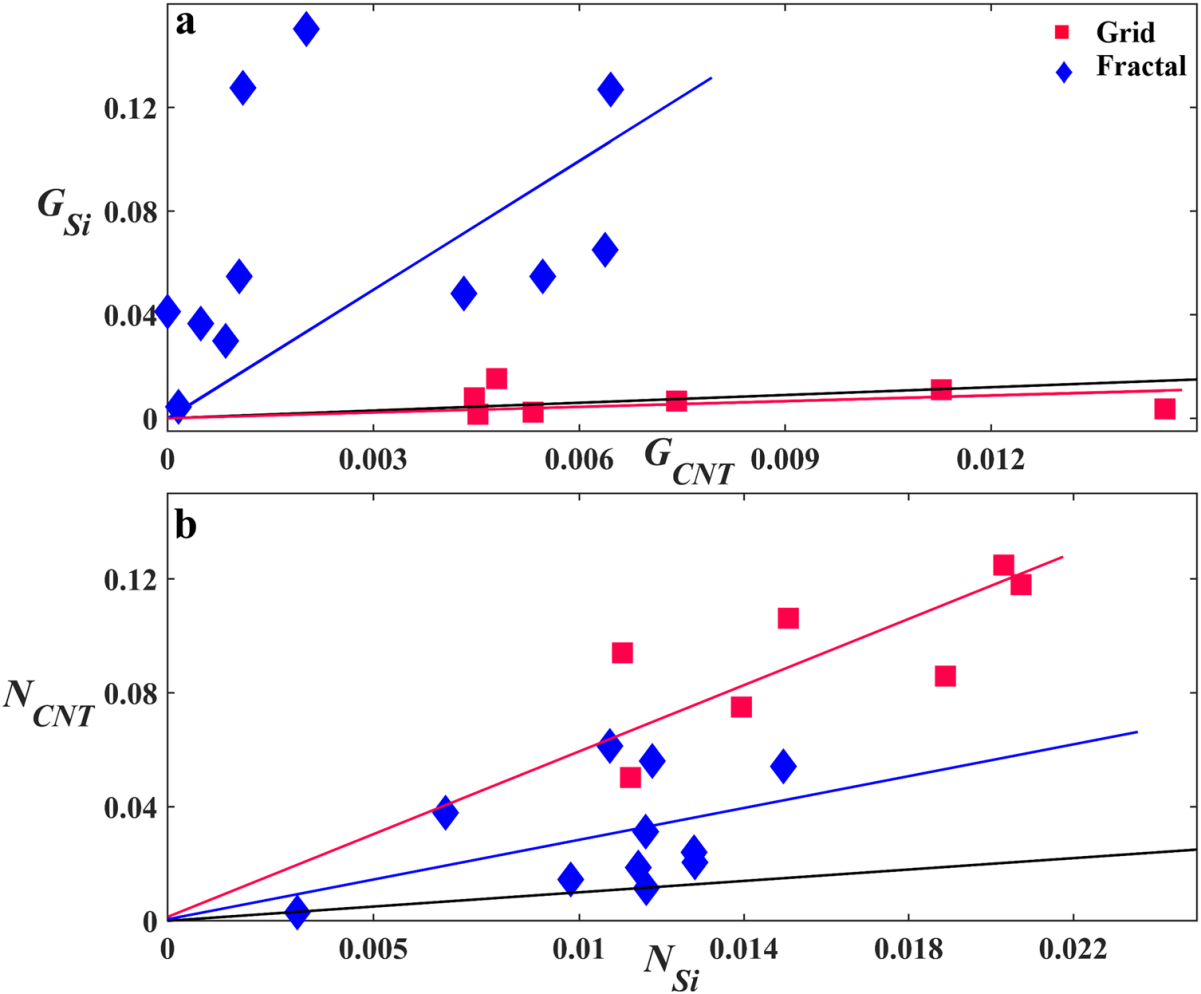
Study of the relationship of *G*_*Si*_ with *G*_*CNT*_ and *N*_*CNT*_ with *N*_*Si*_ for the grid and fractal electrodes. (**a**) Scatterplot of *G*_*Si*_ versus *G*_*CNT*_ for the grids (red) and fractals (blue). (**b**) Scatterplot of *N*_*CNT*_ versus *N*_*Si*_ for the grids (red) and fractals (blue). The solid black lines represent the *G*_*Si*_ = *G*_*CNT*_ and *N*_*CNT*_ = *N*_*Si*_ conditions in (**a**) and (**b**), respectively. The solid red and blue lines are fits through zero for the grids and fractals, respectively.

Having assessed the effect of the SiO_2_ and VACNT regions on the glial and neuronal distributions for each electrode design separately, we then compared the success of the two electrodes in achieving the desired cell distributions (i.e. concentrating neurons and glia in the VACNT and SiO_2_ regions, respectively). Figure 4a and b show scatterplots of *G*_*Si*_ versus *G*_*CNT*_ and *N*_*CNT*_ versus *N*_*Si*_ for the grid and fractal electrodes. The black lines represent the conditions *G*_*Si*_ = *G*_*CNT*_ and *N*_*CNT*_ = *N*_*Si*_. All fractals successfully achieved the condition *G*_*Si*_ > *G*_*CNT*_, while only 2 out of 7 grids did so. On the other hand, all the grids were successful in achieving the condition *N*_*CNT*_ > *N*_*Si*_, whereas 9 out of 11 fractals were successful in doing so. The solid red and blue lines are fits through zero for the grids and fractals and are included as guides to the eye. Although these linear guides are useful for comparing the data to the *G*_*Si*_ = *G*_*CNT*_ and *N*_*CNT*_ = *N*_*Si*_ conditions (represented by the slopes of the black lines), we are not using these fits to imply a strictly linear behavior (the R^2^ values are equal to 0.06 for the grid and 0.32 for the fractal *G*_*Si*_ versus. *G*_*CNT*_ fits, and 0.71 for the grid and 0.41 for the fractal *N*_*CNT*_ versus. *N*_*Si*_ fits).

**Figure 5:**
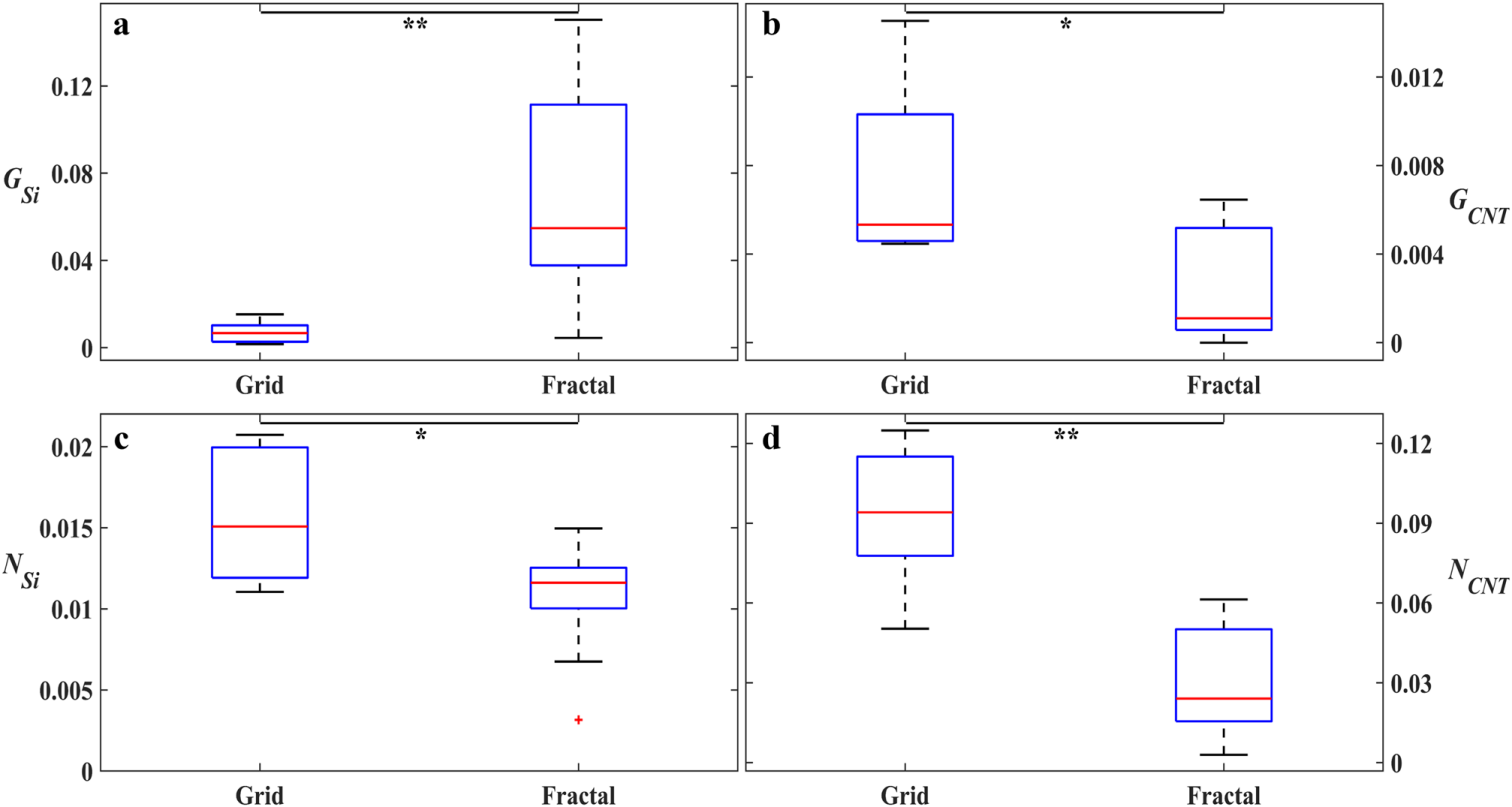
Comparison between the grid and fractal electrodes at 17 DIV in terms of the glial and neuronal behavior on the SiO_2_ and VACNT surfaces. Statistical analysis showing boxplots of (**a**) *G*_*Si*_, (**b**) *G*_*CNT*_, (**c**) *N*_*Si*_, and (**d**) *N*_*CNT*_ between the grid and fractal electrodes. Stars in all panels indicate the degrees of significance: ** and *denote *p* ≤ 0.01 and *p* ≤ 0.05, respectively. The red plus in panel (**c**) is an outlier.

Finally, the grid and fractal electrodes were compared directly for each of the four parameters (*G*_*Si*_, *G*_*CNT*_, *N*_*Si*_, and *N*_*CNT*_). In terms of glial behavior, results of statistical comparisons confirmed that the fractals had significantly higher *G*_*Si*_ than the grids (*p* = 0.0018, Fig. 5a), while the grids had significantly higher *G*_*CNT*_ than the fractals (*p* = 0.0164, Fig. 5b). Considering neuronal behavior, the grids had significantly higher *N*_*Si*_ and *N*_*CNT*_ compared to the fractals (*p* = 0.0333 and 0.0013, respectively, Fig. 5c,d). The fractal outlier in Figs. 3d and 5c has the lowest *N*_*Si*_ likely due to it having the lowest *G*_*Si*_ value among all fractals (this low *G*_*Si*_ was due to variations across different electrodes within a culture).

## Discussion

Our experiments relied on the well-established behavior that glia accumulate on smooth rather than textured surfaces^31,44,45,48^. Whereas most previous studies investigated pure glial cultures on substrates made of a single material featuring different textures, here we focused on retinal neuron-glia co-cultures on a multi-material system (smooth SiO_2_ and textured VACNT) to provide confirmation that different cell types within a co-culture could be made to accumulate on different regions through manipulation of surface texture^31^. To achieve this, we combined micron-scale lateral patterning of the VACNTs (with an average height of 25 µm) with their surface nano-roughness effects, in contrast to previous studies that used unpatterned nano-rough surfaces^32,45,49^, nano-ripples and micro-grooves^46^, and nanowires^31^. We found that, as expected, the observed cell morphologies on the two surfaces were consistent with those observed in the previous textural studies^32,59^. However, the large VACNT heights were shown to be an important factor in our system design—glia residing on the SiO_2_ never extended processes over the VACNT surfaces. As such, the VACNT electrodes acted as barriers to the glia and therefore guided glial coverage across the SiO_2_ surface. Considering the impact of gap size on glial behavior, the smallest gap size in the fractal design, *W*_*Si-min*_, did not prevent glial coverage from extending between the fractal gaps, as suggested by the extended morphology of the glia located in the *W*_*Si-min*_ gaps (Fig. 2d, Supplementary Fig. S4). The smallest SiO_2_ regions within the fractal electrode were therefore connected to increasingly larger areas, providing the glia with the freedom to expand their coverage across these large regions (Supplementary Fig. S4). Although the size of the grid chambers matched the *W*_*Si-min*_ of the fractal electrodes, the disconnected character of the grid electrodes prevented glia that resided in one chamber from accessing other regions of the electrode (Fig. 2c). This left regions within the grid electrode devoid of glia and resulted in the grids having significantly lower *G*_*Si*_ values than the fractal electrodes (Fig. 5a). This may have significant negative effects on the survival and function of neurons in the long term since it is well-known that glia act as the neurons’ life support system^80,81^ and their presence significantly improves synaptic connections between neurons^82^.

Considering neuronal behavior on the SiO_2_ surfaces, neurons rely on surface adhesion for their development and survival. Due to their closer proximity to the VACNT sidewalls, neurons in the grid chambers had a higher chance of adhering to and growing their processes along the electrode edges than those in the larger fractal gaps. This attraction of processes to the sidewalls rather than to the SiO_2_ surfaces could have been further encouraged by chemical signals (neurotrophic factors)^83–86^ from neurons and glia on the VACNT surfaces, potentially lowering *N*_*Si*_ for the grids compared to the fractals. However, we hypothesize that another adhesion-related behavior dominated, causing *N*_*Si*_ to be lowest for the fractals. The strong cell-VACNT adhesion forces experienced by the neurons would have competed with neuron-neuron aggregation forces, slowing down cluster formation in the grid chambers. In contrast, neurons in the fractal gaps would have been less likely to encounter the VACNT edges, resulting in a higher tendency for aggregation of their somas into larger clusters. The presence of glia also promoted this aggregation pattern. During aggregation, processes connecting the larger clusters decreased, with some joining together to form bundles which then contributed to the decrease in *N*_*Si*_ because the process detection algorithm typically counted each bundle as one link between clusters (see “Materials and methods” section). This clustering behavior even left many locations in the fractal gaps completely empty of processes. It is therefore possible that the dominance of this second adhesion-dependent pattern could explain the significantly lower *N*_*Si*_ values for the fractal gaps compared to the grid chambers (Fig. 5c).

Consistent with previous results^48,53^, the VACNT surfaces supported neuron process growth that was larger compared to the SiO_2_ surfaces for both the fractal and grid electrodes (Fig. 3b,d). CNT nano-roughness mimics some of the ECM properties providing guided neuron process growth and better neuron adhesion^41,42^. In addition, they provide favorable mechanical flexibility that can improve neuron process growth and branching^87^, so establishing a suitable environment for neuronal adhesion, survival, and growth without the need of any further chemical surface modification^48^. This resulted in the significantly larger *N*_*CNT*_ than *N*_*Si*_ values for both the grid (Fig. 3b) and fractal electrodes (Fig. 3d). Due to their sensitivity to topographical cues^31,33,88,89^, the processes exhibited a tendency to follow the top and bottom edges of VACNT sidewalls for both electrodes (Fig. 2i,j, Supplementary Fig. S4). The large, connected edge length of both electrode designs provided the opportunity for clusters to form and anchor to the VACNT sidewalls. The larger *N*_*CNT*_ values for the grids when compared to the fractals (Fig. 5d) is likely explained by the higher chances of clusters anchored to the grid’s VACNT sidewalls facilitating greater connections between the processes in the chambers and those on the VACNT surfaces (Fig. 2o and Supplementary Fig. S4).

**Figure 6:**
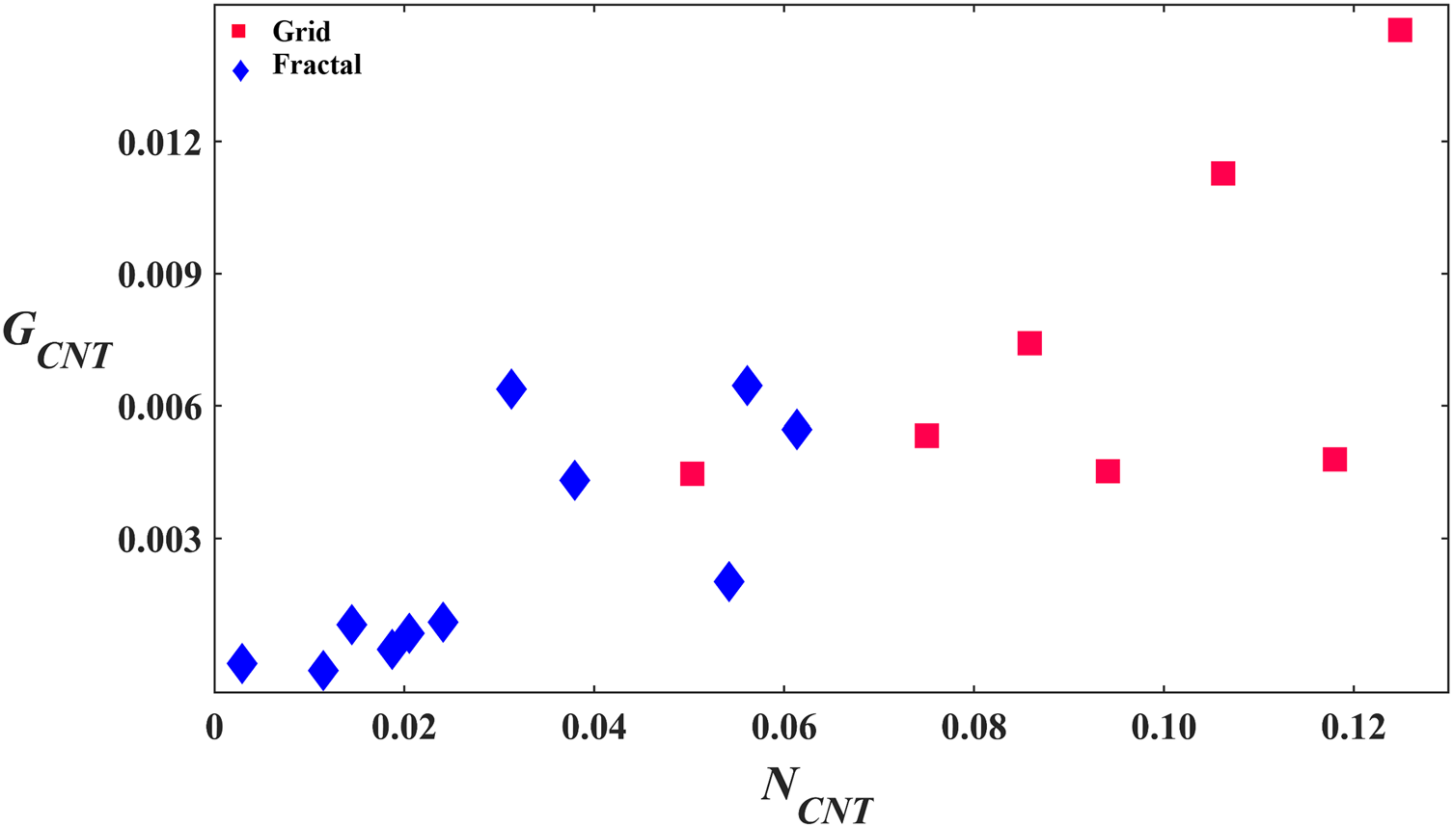
Study of the relationship of *G*_*CNT*_ with *N*_*CNT*_ for the grid and fractal electrodes. Scatterplot of *G*_*CNT*_ versus *N*_*CNT*_ for the grids (red) and fractals (blue).

Now we consider why *G*_*CNT*_ is higher for the grids than the fractals. This is puzzling because the two electrodes have similar *A*_*CNT*_ values and the VACNT surfaces hinder cell division and growth^59^. Based on these considerations, we would expect *G*_*CNT*_ to be similar for the two electrodes. Although speculative, the grid’s neuron-rich VACNT surfaces might have shifted the fate of some of the existing retinal progenitor cells in the environment^90,91^ towards becoming glia. It is known that, in vivo, neuronal stem/progenitor cells have the capability to differentiate into different neural cell types^92^ depending on the physical, biochemical, and topographical cues present in their environment^93^ and perhaps this effect extended to our in vitro environment^62,94^. Accordingly, the larger *N*_*CNT*_ values for the grids over the fractals (Fig. 5d) might have induced their larger *G*_*CNT*_ values (Fig. 5b). This relationship between neurons and glia on the VACNT surfaces of the grids is further suggested by Fig. 6.

Returning to Fig. 5a and b, the smaller values of *G*_*Si*_ and larger values of *G*_*CNT*_ for the grids compared to fractals combine to generate the lack of significant difference between the two parameters in Fig. 3a. In contrast, the larger values of *G*_*Si*_ and smaller values of *G*_*CNT*_ in Fig. 5a and b lead to the significant difference for the fractals seen in Fig. 3c. Although *N*_*CNT*_ is significantly larger than *N*_*Si*_ for both the grids and the fractals, the difference is larger for the grids (Fig. 3b,d). These characteristics can also be seen in Fig. 4. The fractal data lie higher above the black line than the grid data in Fig. 4a and the converse is true for Fig. 4b. The observed increase in *N*_*CNT*_ with *N*_*Si*_ in Fig. 4b is consistent with the above hypothesis of clusters anchored to the VACNT sidewalls mediating connections through processes between neurons in the chambers and those on the VACNT surfaces.

In terms of the underlying aim of accumulating glia on the SiO_2_ surfaces and neurons on the VACNT surfaces, the fractal electrodes performed better at the former and the grid electrodes at the latter. However, the relative lack of nearby glia for the neurons on the grid VACNT surfaces is expected to have detrimental impacts on their survival and electrical activity in the long term. Although the aim of the current study was to consider grid chambers on the scale of the individual glial cell bodies, it is informative to consider the impact of increasing the chamber size. If larger grids were to be fabricated, some beneficial effects for glial surface coverage might be achieved. However, although larger chambers might offer an increased physical freedom for glial coverage due to their less-restricted geometry, there would be an accompanying reduction in proximity to the VACNTs. This would have multiple negative impacts: (1) neurons and glia benefit from chemical cues^83,84^ from each other and both cell types thrive when in close proximity. Implementing grids with larger chambers will decrease this proximity between glia in the middle of the chambers and neurons on the VACNT surfaces, (2) the glia in the middle of the chambers will attract more neurons away from the VACNT sidewalls. This increases the distance between neurons in middle of the chamber and those anchored at the sidewalls (which mediate connections across the two surfaces) which might reduce *N*_*CNT*_, (3) for very large chamber sizes, the probability of having gap areas devoid of cells increases^59^ which results in waste of space in the electrode design, (4) the reduced spatial density of VACNTs will provide less textured surface to support and stimulate the neurons. Supplementary Fig. S5 compares the fractal design to grids featuring a range of chamber sizes and calculates two geometric parameters to explore the theoretical balance between gap connectedness and gap proximity achieved by the two electrode designs. Future experiments should build on this initial modeling to confirm the favorable properties of the fractal over a range of grid sizes.

**Figure 7:**
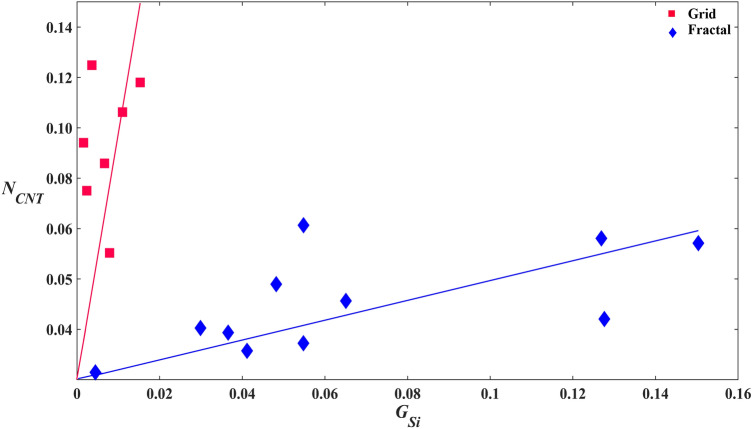
Study of the relationship of *N*_*CNT*_ with *G*_*Si*_ for the grid and fractal electrodes. Scatterplot of *N*_*CNT*_ versus *G*_*Si*_ for the grids (red) and fractals (blue). The solid red and blue lines are fits through zero for the grids and fractals, respectively.

Having established superior glial coverage in the gaps for the fractal electrodes, we now move on to discuss the amount of glia needed to keep neurons healthy and functional on the VACNT electrodes. In order to study the correlation between the glia inside the SiO_2_ gaps and neurons on the VACNT electrodes, we plotted *N*_*CNT*_ versus *G*_*Si*_ in Fig. 7. Considering the fractal design, *N*_*CNT*_ increases with *G*_*Si*_ with the shown linear fit described by an R^2^ of 0.63. We are not using this fit to imply a strictly linear behavior but instead to highlight the following key observations. Firstly, the data trend suggests that an absence of glia (i.e. *G*_*Si*_ = 0) impedes growth of neuron processes substantially. However, the data does not reveal a distinct lower limit (i.e. a *G*_*Si*_ value below which *N*_*CNT*_ falls to zero). This is backed up by qualitative observations showing that there are some VACNT regions that support processes even in the absence of nearby glia. Secondly, when variations in *G*_*Si*_ within the culture result in more glia, this increased presence promotes neuronal growth. Whereas it is possible that further increases in *G*_*Si*_ might eventually cause *N*_*CNT*_ to saturate or even show a depletion^95,96^, our 17 DIV fractal system operates within a regime in which there is no upper limit—the more glia the better. By comparing variations within the fractal electrode, neuron process growth can be taken as an indicator of health because all other geometric factors are constant. We stress that this assumption cannot be extended to comparisons across the two electrode geometries. In particular, the higher *N*_*CNT*_ values achieved by the grids compared to the fractals arises from the geometric factors discussed earlier (i.e. the sidewalls facilitating greater connections across the SiO_2_ and VACNT regions) along with the grid’s larger *G*_*CNT*_ values. For completeness, we include the linear fit for the grids but note that the grid’s significantly lower *G*_*Si*_ values exclude meaningful observations regarding the relationship between *N*_*CNT*_ and *G*_*Si*_.

The above discussion focuses on the relative amounts of glia that are favorable for our system rather than declaring absolute values for broader systems. Previous studies highlight that populations of cell types and consequently ratios of neurons to glia can vary widely between the strains of the same mammalian species^97^ and across subregions of the same structure as a function of neuronal density^9^. With the current understanding of the glia-neuron ratio, it is challenging to determine a lower or upper limit for the number of glia needed in the gaps to guarantee the health and survival of the neurons on the VACNT electrodes. Previous studies claimed that a minimal glial occupation is necessary for protecting neurons from death but did not quantify the degree of occupation necessary^95^. We also acknowledge that the glial coverage and cell arrangements reported in our in vitro system are vastly different to the glial coverage and cell distributions in a gliotic nervous tissue affected by neurodegenerative conditions^15,81^ or insertion of an implant^14,15^. A deeper understanding of the appropriate glia to neuron ratio requires future experiments that would use in vivo models of degenerative retinas such as macular degeneration.

Finally, we compare our current investigations of the grid and fractal electrodes to previous studies of these two geometries. Starting with grids, various formations have featured in glial investigations. The 1978 study discussed earlier provided an initial demonstration that glial cell-free grid chambers remained so throughout the experiment^65^, in spite of negligible height differences between the grid chambers and their surrounding coated areas. The grid electrodes in our studies have much taller walls compared to the 1978 study. This emphasizes the enhanced restricting role of the electrode walls compared to the restrictive role of adherent and non-adherent surfaces. In terms of size dependences, studies have shown an increase in glial coverage as a function of increased surface area within a grid array using chamber sizes ranging from 75 to 200 µm^98^. For grid patterns defined by trenches^64^, the largest trench spacing of 500 µm provided the highest glial coverage. These findings emphasize glial preference for geometries with more degrees of spatial freedom. For example, the trenches can be thought of as being equivalent to the connected gaps in our study and, accordingly, the glia accumulated in them. These findings were therefore consistent with our discussion of physical freedom promoting glial coverage. Other studies investigated the interaction of neurons with grid patterns of negligible heights^34,63^. These showed that neurons followed the patterns and only deviated from them in longer cultures. In the case of our studies, the height of the electrodes can potentially provide more robust confinement of neurons and their processes to the electrode surface for longer times. In another study, hippocampal neurons and glia were shown to colocalize on grid patterns with negligible heights in 26 DIV cultures^62^. The goal of this study was fundamentally different to ours where we aim to guide the two cell types into different regions of the electrode while keeping them in close proximity to each other. Whereas most of the previous studies discussed here focused on pure glial or neuron cultures, we investigated a co-culture of neurons and glia to observe their behavior in more similar conditions to in vivo tissue. Although shaped by a grid-design, these studies used chemical approaches to guide cells to the desired areas. These approaches may not be stable enough for in vivo applications and are fundamentally different to our current study in which the gaps offer the potential for cells to expand their coverage through growth and cell division.

Similarly, the choice of the fractal design as a representative of an ‘open’ geometry is based on previous experimental attention^59^. Whereas the current study focused on the ‘open’ versus ‘closed’ aspect of gap connectedness, in a recent complementary study we examined the multi-scaled character of the fractal gaps and varied the two central fractal parameters, *D* and *m*. Increasing *D* reduced the rate at which the gaps expanded, leading to relatively small gaps with high glial coverages due to their close proximities to the neuron-rich electrodes^59^. Although physically ‘open’, all the gap widths in the fractals for the *m* = 6 design were sufficiently narrow to restrict cell expansion. In contrast, the glial coverage increased for *m* = 4 and 5 to the levels revealed in the current study due to the larger *W*_*Si-min*_ values creating an ‘open’ system. The employment of the *D* = 2, *m* = 5 parameters in the current study was therefore chosen to ensure an ‘open’ system for comparison with the ‘closed’ grid.

## Conclusions

In this study, we used fluorescence microscopy to investigate the fundamental behavior of mouse retinal neurons and glia as they interacted with electrodes in an in vitro environment for 17 DIV. The study demonstrated the importance of electrode geometry in combination with the electrode material properties for controlling the glial response and associated neuronal behavior. By growing VACNTs on a SiO_2_ substrate, we generated laterally patterned regions of textured (VACNT) and smooth (SiO_2_) surfaces. Based on previous studies of cell responses to differing surface textures, the smooth SiO_2_ regions were introduced into the electrode design to predominantly accumulate glia while the role of the textured VACNT regions was to accumulate neurons. Following this strategy, we demonstrated that an ‘open’ system consisting of connected SiO_2_ gaps with multiple scales in a fractal design encourages glial coverage when compared to a ‘closed’ system featuring an array of disconnected gaps in a grid. In contrast, the grid electrode performed better at accumulating neurons on the VACNT surfaces potentially due to the proximity of the neurons to the VACNT sidewalls. However, the relative lack of nearby glia for the neurons on the grid VACNTs is expected to have detrimental impacts on their survival and electrical activity^95^.

The current study was not intended to be a comprehensive investigation of various possible geometries, but rather had the aim of demonstrating some fundamental behaviors for informing future designs by comparing two examples. Our choice of grid and fractal electrodes as the representatives of ‘closed’ and ‘open’ systems builds on previous studies of these geometries in terms of both applications and fundamental cell behavior. Based on our experimental comparisons, future electrode designs should include carefully balancing gap connectedness and proximity. We have presented two possible parameters for quantifying these effects in the Supplementary Information and have discussed how the connectedness of the fractals combined with relatively large proximity gives the cells higher chances of survival compared to the grids.

Based on these results, we hypothesize that there exists an optimized combination of material and geometry that will maximize the positive responses of different cell types within the tissue interacting with an electrode. As an example, the current fractal pattern can be modified through branch elimination or rotation to remove barriers in the electrode’s central gap region while connecting the pattern to a boundary rectangle to maintain a continuous electrode. Such strategies increase the connectedness and accessibility of the SiO_2_ gaps for the glia while keeping their proximity to the VACNT branches approximately the same (Supplementary Fig. S6a,b). In contrast to these positive modifications, introducing extra branches into the fractal design to increase proximity can lead to negative consequences, in particular creating closed SiO_2_ regions that reduce the connectedness of the geometry (Supplementary Fig. S6c). In the long term, this optimization could be used to maximize the efficiency of implantable devices for neuronal recording and stimulation.

Although our study demonstrates the fundamental ability to control spatial distributions of glia and neurons, future experiments will need to confirm the benefits in terms of neuron health and their electrical stimulation. Considering health consequences, the current fractal electrodes operated within a regime in which the increased presence of nearby glia correlated with enhanced growth of neuron processes on the electrodes, pointing to a ‘the more glia the better’ approach for neuron health. We caution that this operational regime is not expected to be universal and that upper limits of glial accumulation might need to be identified and quantified for some systems. In particular, although our in vitro studies represent a controlled model for in vivo behavior, differences between these environments will need to be accommodated in the long-term. For example, in vivo experiments will involve electrode interactions with a three-dimensional structurally intact neural tissue. Glia will then be present in the electrode layer (i.e. accumulating in the gaps between the 25 µm high electrodes) and in the retinal layers above the electrode. Practically, we note that the implanted electrode will interface with unhealthy retinal tissue that is already showing signs of reactive gliosis and is remodeled compared to a healthy tissue^99,100^. Therefore, the excessive glia in the three-dimensional space above the electrode will need to be guided away from the VACNT surfaces serving as the neuron-electrode interface. Guiding the glia into the electrode’s connected gaps will attract more neurons to the surface of the electrode^99^. The relative health contributions of the glia in the electrode gaps and those in the retinal layer above the electrode forms an interesting question. However, future electrodes are expected to assume a more three-dimensional character using higher aspect ratio VACNTs, allowing the electrode to penetrate deeper into the neural tissue and be closer to the targeted neurons. The interface layer will then play an increasing role for these future electrode designs.

Considering electrical stimulation, previous studies have demonstrated the high potential of CNTs as a material system. Both unpatterned and patterned CNTs have been electrically biased to stimulate^50,101^, record^51,102^, and even boost electrical signaling in neurons^51,103^. However, these previous studies did not investigate the impact of cell arrangement. Future studies will need to investigate the degree to which the favorable stimulation properties highlighted in the previous studies can be enhanced through the arrangement of networks of cells presented in our study. Here, we employed large-scale electrodes to explore the holistic arrangement of the cells. Their translation into brain or retinal stimulating implants would need to accommodate higher resolutions and here we consider several potential approaches (Supplementary Fig. S7). The simplest translation would be to employ the fractal electrode as the active electrode bounded by a rectangular ground electrode featuring a gap to allow passage of the active electrode. However, the active electrode would need to be scaled down or subdivided into component electrodes to match the sizes of typical brain implants. A more appealing approach, applicable to retinal implants, would use the large-scale fractal as the ground electrode with an array of small holes defined along its branches. Each hole would then encapsulate an active square electrode deposited on a photodiode layer that is separated from the ground electrode by an insulator (see references^56,57^ for simulations of this photodiode operation). Future research will focus on replacing the square active electrodes with fractal electrodes to potentially encourage process growth and also to increase their electrical capacitance and hence ability to stimulate the neurons^57,58^. In this approach, the SiO_2_ gaps for accommodating the glia would come at the expense of not having a highly dense array of active electrodes. However, they will ensure a healthier neuron-electrode interface. If the general principles shown in the current study extend to in vivo implants, they will ensure that large numbers of neurons reside within the electrodes’ stimulating fields and that their prolonged health provided by the nearby glia will result in operating stability.

## Supplementary Information

Supplementary Information.

## Data Availability

All the data and the related code is available at: https://doi.org/10.6084/m9.figshare.20044022.
