# Supplementary material for: Comparison of fractal and grid electrodes for studying the effects of spatial confinement on dissociated retinal neuronal and glial behavior
Source: Sci Rep. 2022 Oct 20;12:17513. doi: 10.1038/s41598-022-21742-y (PMC9584887; doi:10.1038/s41598-022-21742-y)
Supplement: Supplementary file 1 — Supplementary Information. [file 41598_2022_21742_MOESM1_ESM.docx]

**Comparison of fractal and grid electrodes for studying the effects of spatial confinement on dissociated retinal neuronal and glial behavior**

Saba Moslehi^1,2^, Conor Rowland^1,2^, Julian H. Smith^1,2^, Willem Griffiths^3^, William J. Watterson^1,2^, Cristopher M. Niell^3,4^, Benjamín J. Alemán^1,2,5,6^, Maria-Thereza Perez^7,8^ and Richard P. Taylor^*1,2,6^

^1^ Physics Department, 1371 University of Oregon, Eugene, OR, 97403, USA

^2^ Materials Science Institute, 1252 University of Oregon, Eugene, OR, 97403, USA

^3^ Department of Biology,1210 University of Oregon, Eugene, OR 97403, USA

^4^ Institute of Neuroscience, 1254 University of Oregon, Eugene, OR 97403, USA

^5^ Oregon Center for Optical, Molecular and Quantum Science,1274 University of Oregon, Eugene, OR, 97403, USA

^6^ Phil and Penny Knight Campus for Accelerating Scientific Impact, 1505 University of Oregon, Franklin Blvd. Eugene, OR, 97403, USA

^7^ Department of Clinical Sciences Lund, Division of Ophthalmology, Lund University, SE-221 84 Lund, Sweden

^8^ NanoLund, Lund University, SE-221 00 Lund, Sweden

*Email: [rpt@uoregon.edu](mailto:rpt@uoregon.edu)

**Supplementary Information**

**1. Fractal and grid generation and quantification**

H-Tree fractals are generated through repetition and scaling of H patterns. The first order H is constructed from 2 horizontal branch segments (length *L_0_* – zeroth order branch segment) and 4 perpendicular branch segments (length *L_1_* – first order branch segment), where *L_1_* depends on *L_0_* through the fractal dimension *D* (Supplementary Fig. 1):

$$L_{1}=\frac{L_{0}}{2^{\frac{1}{D}}}$$

Considering the recursive relationship between the consecutive branch segments, the *n*^th^ order branch segment length is then given by:

$$L_{n}=\frac{L_{0}}{2^{\frac{n}{D}}}$$

The total fractal edge length, *E_f_* , for a fractal with *N* repeating branches is given by:

$$E\left( N,W,D,W_{CNT} \right)=\frac{2A_{CNT}}{W_{CNT}}+W_{CNT}\times2^{(N+1)}-\sum_{n=1}^{N} W_{CNT}\times2^{n}$$

where *W* and *W_CNT_* are the overall and branch electrode widths respectively. *A_CNT_*, the surface area covered by all the VACNT branches, is given by:

$$A_{CNT}(N,W,D,W_{CNT})=W_{CNT}L_{f}-\sum_{n=1}^{N} 2^{n}({W_{CNT}^{2}}/2)$$

And *L_f_*, the total length of the branches is:

$$L_{f}\left( N,W,D,W_{CNT} \right)=\left( \frac{W-W_{CNT}}{2\sum_{n=0}^{\frac{(N-1)}{2}} 1/{2^{\frac{2n}{D}}}} \right)\sum_{n=0}^{N} {2^{n+1}}/{2^{\frac{n}{D}}}$$

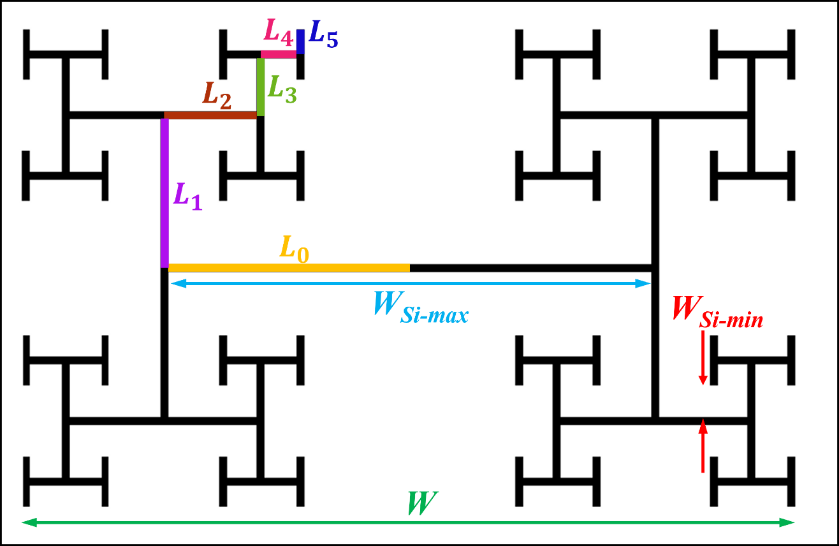


**Supplementary Figure 1.** A schematic of a H-Tree fractal featuring *m* = 3 repeating levels of the H pattern. *L_0_*, *L_1_*, *L_2_*, *L_3_*, *L_4_*, and *L_5_* are the lengths of the 0^th^ to 5^th^ order branches. The fractal’s *W_Si-min_*, *W_Si-max_* and total width *W* are marked as well.

To quantify the sizes of the fractal’s gaps, we consider the following two feature lengths which correspond to smallest and largest separation distances between branches measured in either the horizontal or vertical directions (Supplementary Fig. 1):

$$W_{Si-min}\left( N,W,D,W_{CNT} \right)=L_{0}\left( N,W,D,W_{CNT} \right)\left( 1/{2^{\frac{N-2}{D}}}-1/{2^{\frac{N}{D}}} \right)-{W_{CNT}}/2$$

$$W_{Si-max}=2L_{0}$$

Where *L_0_* is the length of the zeroth order branch of the fractal:

$$L_{0}\left( N,W,D,W_{CNT} \right)= {(W-W_{CNT})}/{2\sum_{n=0}^{\frac{(N-1)}{2}} 1/{2^{\frac{2n}{D}}}}$$

We also calculated the maximum connected gap area, *A_c_* for the fractal (defined as the maximum connected area in the gaps not surpassing the edge of the electrode):

$$A_{c}\left( N,W,D,W_{CNT} \right)=2\left( \frac{W-W_{CNT}}{2\sum_{n=0}^{\frac{(N-1)}{2}} 1/{2^{\frac{2n}{D}}}} \right)^{2}\left( \sum_{n=0}^{{(N-1)}/2} 1/{2^{\frac{2n+1}{D}}}-\sum_{n=1}^{{(N-1)}/2} \sum_{m=n}^{{(N-1)}/2} 1/{2^{\frac{2m+2n+1}{D}}} \right)-\left( \frac{W-W_{CNT}}{2\sum_{n=0}^{\frac{(N-1)}{2}} 1/{2^{\frac{2n}{D}}}} \right)W_{CNT}\left( \sum_{n=1}^{{(N-1)}/2} \sum_{m=2n+1}^{N} {2^{m}}/{2^{2n+\frac{m}{D}}}+\sum_{n=0}^{N} 1/{2^{\frac{2n}{D}}} \right)$$

For a grid with square chambers, an overall width, *W*, number of chambers in a row, *M*, and electrode width, *W_CNT_*, VACNT surface area, *A_CNT_* is given by:

$$A_{CNT}\left( W,M,W_{CNT} \right)={(M+1)(2WW}_{CNT}-(M+1)W_{CNT}^{2})$$

The grid chamber width is then given by:

$$W_{Si-min}\left( W,M,W_{CNT} \right)=\frac{W-(M+1)W_{CNT}}{M}$$

Adopting the width measurement criteria of the fractal electrode, *W_Si-max_* = *W_Si-min_*.

*A_c_* would then be the area of a single chamber:

$$A_{c}=W_{Si-min}^{2}$$

The total edge length of the grid is:

$$E\left( W,M,W_{CNT} \right)=4(M+1)(W-M)W_{CNT}$$

We next consider the distance *d* between each gap pixel and its nearest branch pixel. To calculate *d_mean_* as well as *d_median_* and the associated mean proximity, *P_mean_*, binary masks for the grid and fractal electrodes were generated. *d_mean_* was the mean of all the distance values within the gaps. The proximity matrix was created by assigning the value of *P* = 1/*d* to each gap pixel. *P_mean_* was then calculated by averaging over all matrix elements of *P* in the gaps. We chose to define *d_mean_* and *P_mean_* using the binary masks approach rather than analytical calculations due to the complexity of the fractal geometry. We note the sensitivity of the applied method to the pixel size. We emphasize that this sensitivity would not affect our results because the pixel size is kept constant across the two geometries and we’re interested in relative changes with respect to *A_c_* rather than absolute values.

To investigate the effect of grid chamber size, theoretical grid patterns were generated with chamber widths ranging from the smallest size equaling *W_Si-min_* to the maximum size equaling √*A_c_* for the 2-5 fractal. To generate this set of grids, we set *W_CNT_*, *A_CNT_*, and *E* constant between all of the theoretical grids and the 2-5 fractal. *A_bounding_* and the number of chambers *M* are determined from the equations mentioned above. Having determined the grid width as the √*A_c_* we then solve for the total length of each grid electrode *W* as a function of *M*, *W_Si-min,_* and *W_CNT_*:

$$W_{Si-min}\left( W ,M,W_{CNT} \right)=\frac{W-(M+1)W_{CNT}}{M}$$

$$W\left( M,W_{CNT},W_{Si-min} \right)=MW_{Si-min}+(M+1)W_{CNT}$$

Inserting *W* into the equation for *A_CNT_* and substituting for *L_g_* (total length of the grid) we then solve the equation below for *M*:

$$\left( 2W_{CNT}W_{Si-min}+W_{CNT}^{2} \right)M^{2}+\left( 2W_{CNT}W_{Si-min}{+3W}_{CNT}^{2} \right)M+{2W}_{CNT}^{2}-A_{CNT}=0$$

The chosen *M* for each grid, is then either the floor or ceiling value of the solved *M* that satisfies the *A_CNT_* condition more closely. The parameters for theoretical grids and fractals are summarized in supplementary table 1.

| Electrode | *W_Si-min_*  (µm) | *M* | *m* | *W*  (µm) | *E*  *(µm)* | *d_median_*  *(µm)* | *A_CNT_*  (µm^2^) | *A_Si_*  (µm^2^) | *A_bounding_*  (µm^2^) | *A_c_*  (µm^2^) | *P*  (µm^-1^) |
| --- | --- | --- | --- | --- | --- | --- | --- | --- | --- | --- | --- |
| Grid | | | | | | | | | | | |
| G1 | 60.00 | 43 | N/A | 3.51×10^3^ | 4.66×10^5^ | 9.06 | 5.36×10^6^ | 6.91×10^6^ | 1.23×10^7^ | 3.74×10^3^ | 0.2731 |
| G2 | 100.00 | 32 | N/A | 3.86×10^3^ | 4.25×10^5^ | 14.88 | 4.66×10^6^ | 1.02×10^7^ | 1.49×10^7^ | 10^4^ | 0.1860 |
| G3 | 223.61 | 22 | N/A | 5.38×10^3^ | 4.54×10^5^ | 33.01 | 4.74×10^6^ | 2.42×10^7^ | 2.89×10^7^ | 5×10^4^ | 0.0973 |
| G4 | 316.23 | 18 | N/A | 6.07×10^3^ | 4.34×10^5^ | 46.27 | 4.47×10^6^ | 3.24×10^7^ | 3.69×10^7^ | 10^5^ | 0.0732 |
| G5 | 707.11 | 12 | N/A | 8.74×10^3^ | 4.42×10^5^ | 103.55 | 4.48×10^6^ | 7.20×10^7^ | 7.65×10^7^ | 5×10^5^ | 0.0372 |
| G6 | 1000 | 10 | N/A | 1.02×10^4^ | 4.41×10^5^ | 146.59 | 4.45×10^6^ | 1.00×10^8^ | 1.04×10^8^ | 10^6^ | 0.0277 |
| G7 | 1224.74 | 9 | N/A | 1.12×10^4^ | 4.42×10^5^ | 179.60 | 4.45×10^6^ | 1.21×10^8^ | 1.26×10^8^ | 1.5×10^6^ | 0.0233 |
| G8 | 1414.21 | 8 | N/A | 1.15×10^4^ | 4.08×10^5^ | 207.10 | 4.10×10^6^ | 1.28×10^8^ | 1.32×10^8^ | 2×10^6^ | 0.0206 |
| G9 | 1581.14 | 8 | N/A | 1.28×10^4^ | 4.56×10^5^ | 231.70 | 4.59×10^6^ | 1.60×10^8^ | 1.65×10^8^ | 2.5×10^6^ | 0.0187 |
| G10 | 1732.05 | 8 | N/A | 1.40×10^4^ | 4.99×10^5^ | 253.70 | 5.02×10^6^ | 1.92×10^8^ | 1.97×10^8^ | 3×10^6^ | 0.0173 |
| G11 | 1870.83 | 7 | N/A | 1.33×10^4^ | 4.20×10^5^ | 274.09 | 4.22×10^6^ | 1.71×10^8^ | 1.76×10^8^ | 3.50×10^6^ | 0.0161 |
| G12 | 2014.45 | 7 | N/A | 1.43×10^4^ | 4.52×10^5^ | 295.12 | 4.54×10^6^ | 1.99×10^8^ | 2.03×10^8^ | 4.06×10^6^ | 0.0151 |
| Fractal | | | | | | | | | | | |
| 2-4 | 137.12 | N/A | 4 | 6.26×10^3^ | 2.36×10^5^ | 57.60 | 2.36×10^6^ | 2.54×10^7^ | 2.77×10^7^ | 4.77×10^6^ | 0.0572 |
| 2-5 | 60.00 | N/A | 5 | 6.26×10^3^ | 4.62×10^5^ | 30.94 | 4.62×10^6^ | 2.31×10^7^ | 2.77×10^7^ | 4.06×10^6^ | 0.1091 |
| 2-6 | 25.03 | N/A | 6 | 6.26×10^3^ | 8.83×10^5^ | 11.32 | 8.82×10^6^ | 1.89×10^7^ | 2.77×10^7^ | 3.10×10^6^ | 0.2157 |

**Supplementary Table 1.** Theoretical grid and fractal parameters.

**2. SEM images of the grid and fractal electrodes**


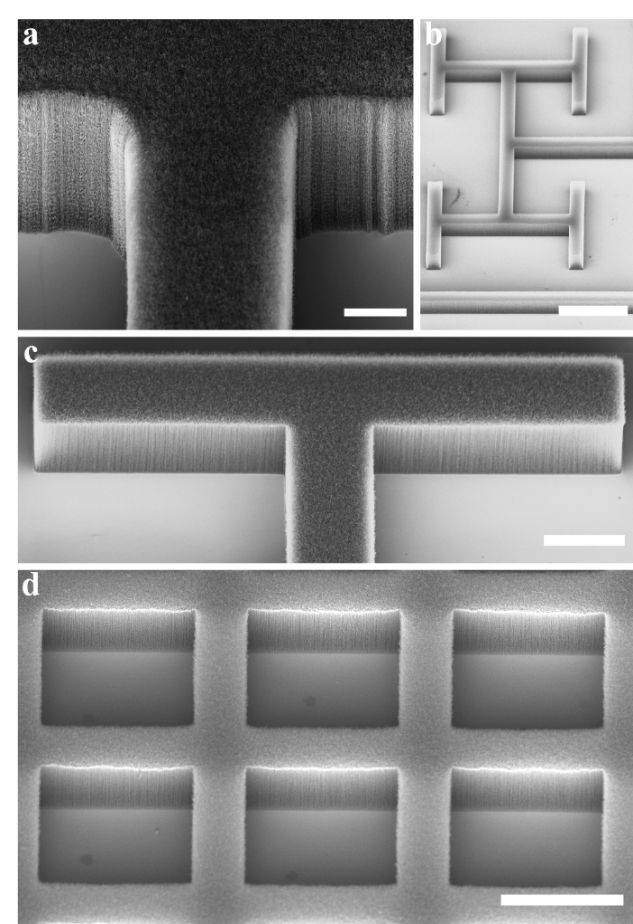


**Supplementary Figure 2.** 40° tilted SEM images of examples of grid and fractal electrodes. (a, b, c) SEM images of a fractal electrode showing the top surface and the sidewalls of the VACNTs, (d) SEM image of a grid electrode showing multiple chambers surrounded by VACNT walls. Scale bars are 10, 100, 20, and 50 µm in (a), (b), (c), and (d) respectively.

**3. Algorithm steps for glia and neuron process detection**


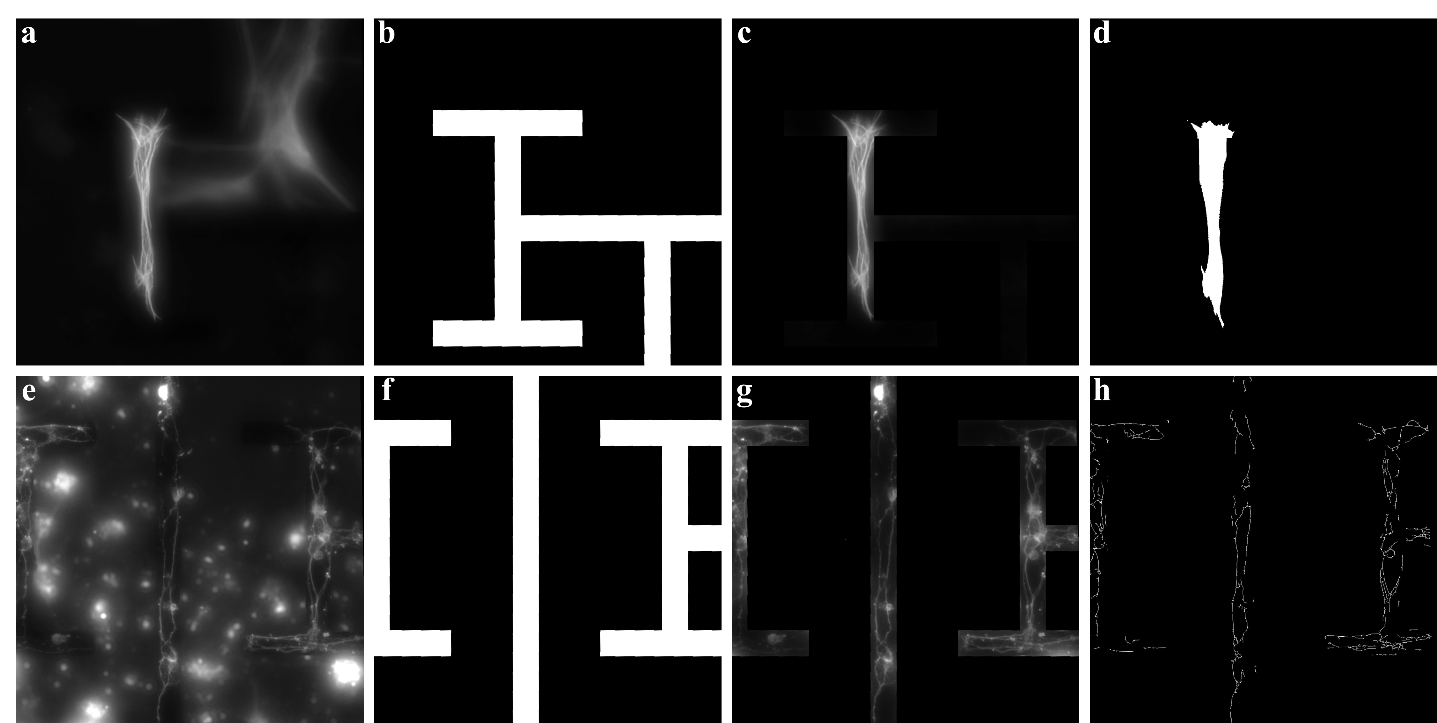


**Supplementary Figure 3.** Representative images of the algorithm steps for glia and neuron process detection and measurements. Fluorescence images of (a) glia and (e) neurons on the VACNT and SiO_2_ surfaces of a fractal electrode. The focus is set on the VACNT surface. (b, f) The binary masks associated with the FOVs shown in (a) and (e) respectively. (c, g) Combination of the FOVs in (a, b) and (e, f) respectively for isolating the glia and neurons on the VACNT surfaces. (d, h) Applying the glia thresholding and neuron process detection MATLAB algorithms to the FOVs shown in (c) and (g).

**4. Fluorescence images of retinal cells interacting with the grid and fractal electrodes**


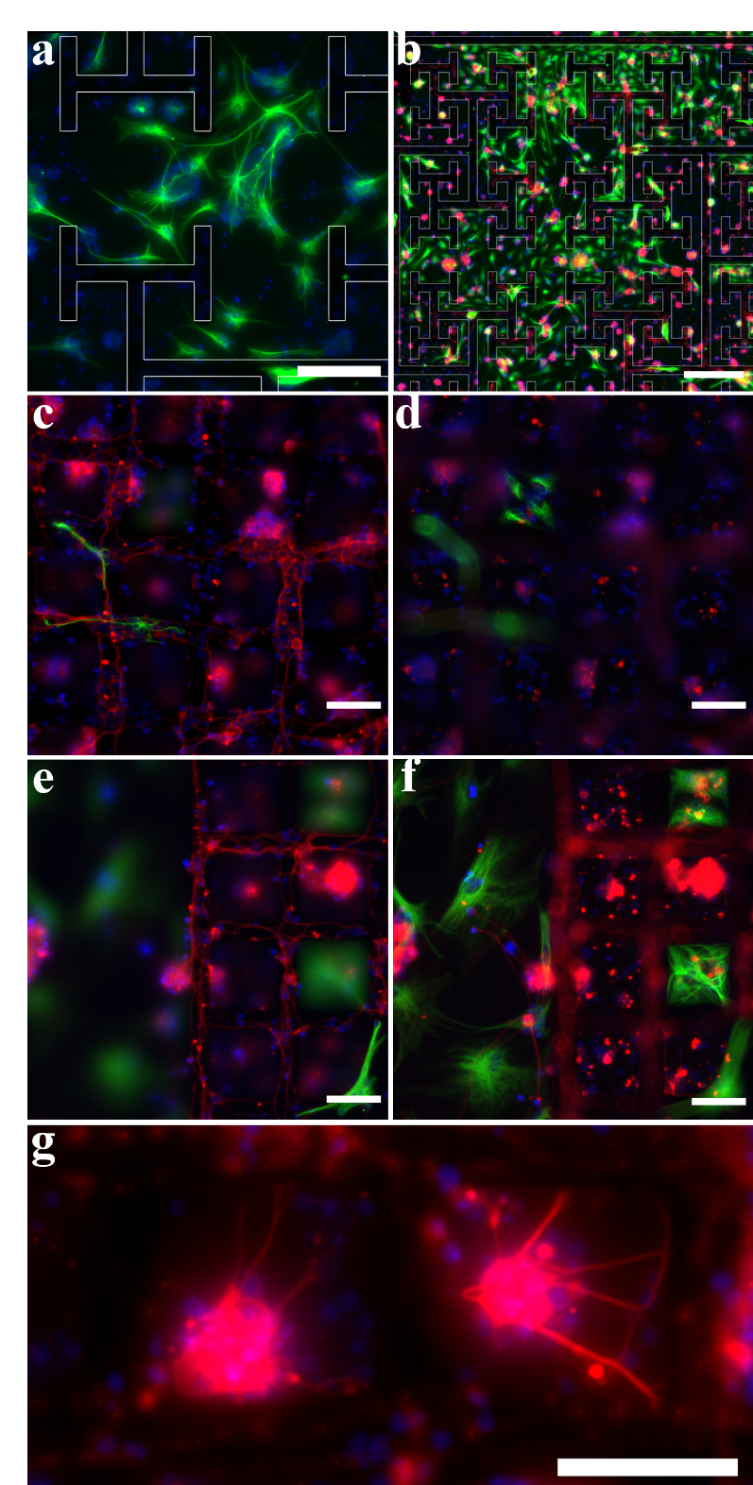


**Supplementary Figure 4.** Representative examples of fluorescence images of retinal cells interacting with the grid and fractal electrodes at 17 DIV (green = GFAP labelled glia; red = β-tubulin III labelled neurons; blue = DAPI labeled nuclei). (a) Glia inside the gaps of a fractal electrode accompanied by cell nuclei. (b) Neurons, glia, and cell nuclei within the gaps of a fractal electrode. The whole gap area is interconnected giving the cells the potential to cover large surfaces. Neurons, glia, and cell nuclei accumulating on the (c) VACNT and (d) SiO_2_ surfaces of a grid electrode. Neurons, glia, and cell nuclei outside and within the patterned area of a grid electrode with the focus being on the (e) VACNT and (f) SiO_2_ surfaces. (g) Cell clusters sending neuron processes towards VACNT sidewalls in two side-by-side chambers of a grid. The images in (c) and (d) show the same FOV at two different focal planes, as do (e) and (f). Scale bars are 100 µm in (a), 200 µm in (b), and 50 µm in (c), (d), (e), (f), and (g).

**5. Interplay between *d_median_* and *A_c_* for experimental and theoretical electrodes**

To better understand the importance of the proximity of SiO_2_ gaps to the VACNT electrodes, the interplay between the median distance *d_median_* and the maximum connected area *A_c_* are plotted in Supplementary Fig. 5 for grid electrodes in which the chambers vary in width from *W_Si-min_* (i.e. the experimentally-investigated grid) to the width that generates chambers with enclosed areas matching the *A_c_* value of the experimental fractal electrode (i.e. the *m* = 5 fractal with *D* = 2). For comparison, we also include hypothetical fractals with repeating levels of *m* = 4 and 6 with *D* = 2. The fractal electrodes provide a large *A_c_* while maintaining a relatively low *d* when compared to the grid electrodes. Compared to the hypothetical grid electrodes, the interconnectedness of the fractal gaps is expected to be more advantageous for the whole system of neuron-glial co-cultures and will potentially regulate better interactions while balancing the need for electrode surfaces that support neuronal attachment and growth. We note that the distributions of *d* values are inevitably large due to the large gap sizes.


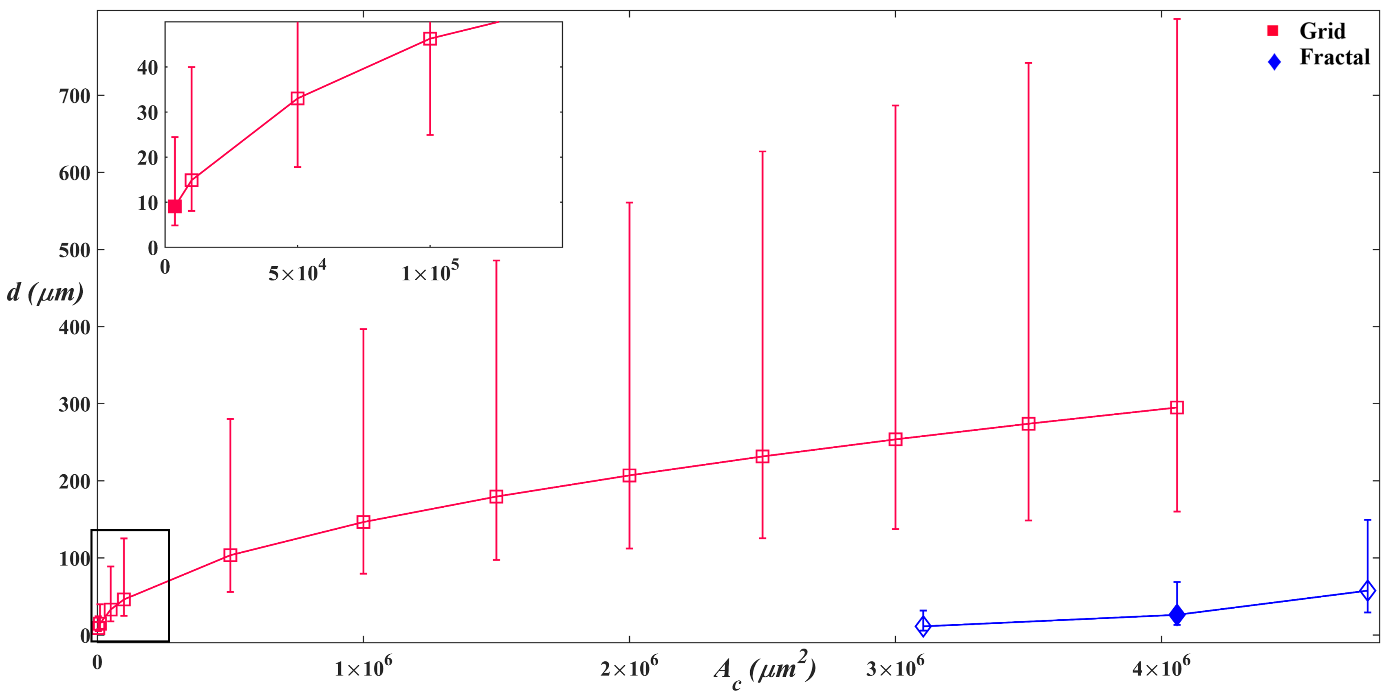


**Supplementary Figure 5.** The median of *d* plotted vs *A_c_* for grid electrodes with different *W_Si-min_* values (increasing from left to right) and fractal electrodes with *D* = 2 and *m* = 4, 5, and 6 (with *W_Si-min_* values increasing from left to right). In each case, the filled symbols represent the electrodes studied experimentally. The error bars are the 25% and 75% quartiles with respect to the median values. The inset is a zoom-in on the black rectangle to clearly distinguish between the first four grid data points.

**6. Optimized electrode patterns**

**
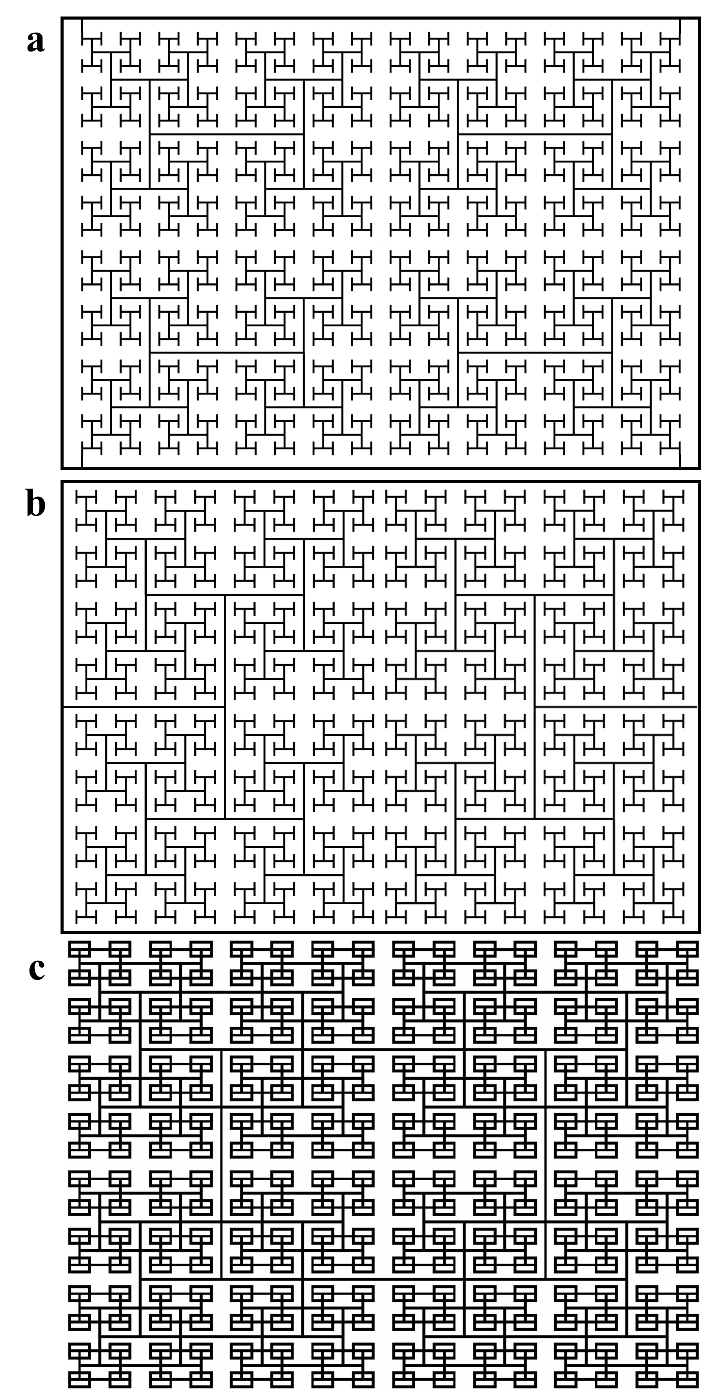
**

**Supplementary Figure 6.** Examples of improved and worsened electrode geometries. (a, b) Improved electrode based on a *D* = 2 and *m* = 5 H-Tree fractal enclosed within a rectangular boundary. For design (a), the first order H has been removed from the tree and the four outer corners of the resulting geometry have been connected to the rectangular boundary. For design (b), the fractal has cut in half through the zeroth order branch segment, each half was then rotated through 180° and attached to the boundary. In this second design, the H-tree branches spread from the boundary inwards rather than spreading out from the origin as occurred in the original fractal electrode used in this study. By removing branches from the central section of the design, both the (a) and (b) modifications create a completely connected SiO_2_ gap region while keeping the proximity of the gaps to the electrodes roughly comparable to the original fractal electrode. (c) Worsened fractal electrode based on a *D* = 2 and *m* = 5 H-Tree fractal. The shown modifications increase the proximity between the gaps and the electrodes but decrease the gap connectedness drastically.

**7. Potential applications of the fractal electrode designs.**

**
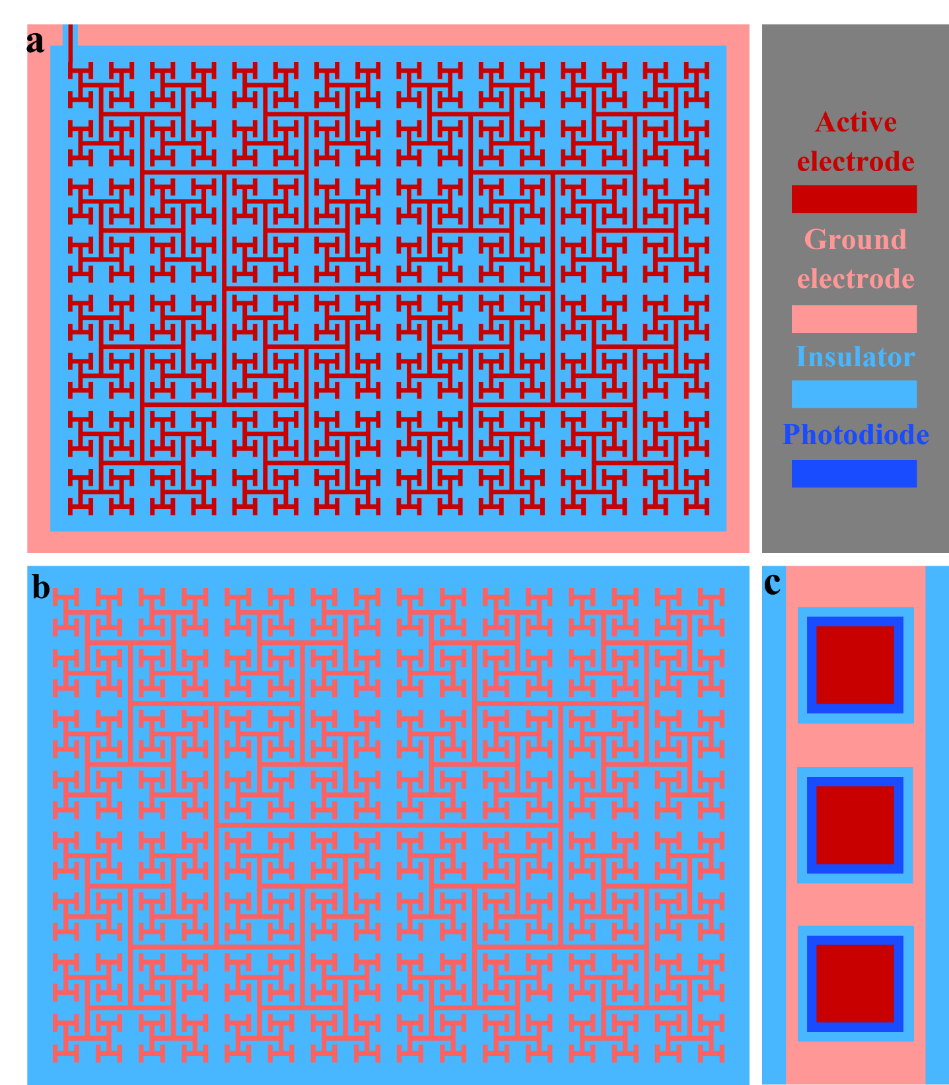
**

**Supplementary Figure 7.** Potential electrode designs for brain and retinal stimulation purposes. (a) For brain stimulation purposes, the large-scale fractal pattern (red) serves as the active electrode bounded by a rectangular ground electrode (pink). (b) For retinal stimulation purposes, the large-scale fractal pattern serves as the ground electrode (pink) with an array of small holes inserted along its branches. (c) The zoom-in on a fractal branch in (b) showing three holes within the branch of the ground fractal electrode. Each hole features an active square electrode (red) above a photodiode layer (dark blue) separated from the ground fractal electrode by an insulating region (light blue).
